# Gene Recruitments and Dismissals in the Argonaut Genome Provide Insights into Pelagic Lifestyle Adaptation and Shell-like Eggcase Reacquisition

**DOI:** 10.1093/gbe/evac140

**Published:** 2022-10-26

**Authors:** Masa-aki Yoshida, Kazuki Hirota, Junichi Imoto, Miki Okuno, Hiroyuki Tanaka, Rei Kajitani, Atsushi Toyoda, Takehiko Itoh, Kazuho Ikeo, Takenori Sasaki, Davin H E Setiamarga

**Affiliations:** Marine Biological Science Section, Education and Research Center for Biological Resources, Faculty of Life and Environmental Science, Shimane University, Okinoshima, Shimane 685-0024, Japan; Graduate School of Science, The University of Tokyo, Bunkyo-ku, Tokyo 113-8654, Japan; Department of Applied Chemistry and Biochemistry, National Institute of Technology (KOSEN), Wakayama College, Gobo, Wakayama 644-0012, Japan; Center for Information Biology, National Institute of Genetics, Mishima, Shizuoka 411-8540, Japan; Division of Microbiology, Department of Infectious Medicine, Kurume University School of Medicine, Kurume, Fukuoka 830-0011, Japan; School of Life Science and Technology, Tokyo Institute of Technology, Meguro-ku, Tokyo 152-8550, Japan; School of Life Science and Technology, Tokyo Institute of Technology, Meguro-ku, Tokyo 152-8550, Japan; Comparative Genomics Laboratory, National Institute of Genetics, Mishima, Shizuoka 411-8540, Japan; Advanced Genomics Center, National Institute of Genetics, Mishima, Shizuoka 411-8540, Japan; School of Life Science and Technology, Tokyo Institute of Technology, Meguro-ku, Tokyo 152-8550, Japan; Graduate School of Science, The University of Tokyo, Bunkyo-ku, Tokyo 113-8654, Japan; Graduate School of Science, The University of Tokyo, Bunkyo-ku, Tokyo 113-8654, Japan; The University Museum, The University of Tokyo, Bunkyo-ku, Tokyo 113-0033, Japan; Department of Applied Chemistry and Biochemistry, National Institute of Technology (KOSEN), Wakayama College, Gobo, Wakayama 644-0012, Japan; The University Museum, The University of Tokyo, Bunkyo-ku, Tokyo 113-0033, Japan

**Keywords:** comparative genomics, biomineralization, shell matrix proteins, concerted evolution, Hox cluster

## Abstract

The paper nautilus or greater argonaut, *Argonauta argo*, is a species of octopods which is characterized by its pelagic lifestyle and by the presence of a protective spiral-shaped shell-like eggcase in females. To reveal the genomic background of how the species adapted to the pelagic lifestyle and acquired its shell-like eggcase, we sequenced the draft genome of the species. The genome size was 1.1 Gb, which is the smallest among the cephalopods known to date, with the top 215 scaffolds (average length 5,064,479 bp) covering 81% (1.09 Gb) of the total assembly. A total of 26,433 protein-coding genes were predicted from 16,802 assembled scaffolds. From these, we identified nearly intact HOX, Parahox, Wnt clusters, and some gene clusters that could probably be related to the pelagic lifestyle, such as *reflectin*, *tyrosinase*, and *opsin*. The gene models also revealed several homologous genes related to calcified shell formation in Conchiferan mollusks, such as Pif-like, SOD, and TRX. Interestingly, comparative genomics analysis revealed that the homologous genes for such genes were also found in the genome of the shell-less octopus, as well as *Nautilus*, which has a true outer shell. Therefore, the draft genome sequence of *Arg. argo* presented here has helped us to gain further insights into the genetic background of the dynamic recruitment and dismissal of genes to form an important, converging extended phenotypic structure such as the shell and the shell-like eggcase. Additionally, it allows us to explore the evolution of from benthic to pelagic lifestyles in cephalopods and octopods.

**Significance**

It has been suggested that the coleoid cephalopod (squids, cuttlefish, and octopus) lineage went through large-scale genome reorganization. To understand the extent of the genome reorganization and its impact on cephalopod genome biology and evolution, the identification of the argonaut genome is the first indication that genome size amplification has not occurred in all octopods. This provides a view of drastic genome shuffling that was previously thought to be specific to cephalopods and essential information for tracing the process. It also demonstrates that short read-based genome assembly is still effective in marine animal genome analysis, which is generally considered difficult.

## Introduction

The paper nautilus, or the argonaut *Argonauta*, is a member of Argonautoidea, a superfamily of octopods (Cephalopoda, Octopodiformes), but with specialized characteristics not found in other octopus species. It is a cosmopolitan species distributed in the global tropical and subtropical open seas (Norman 2000). Phylogenetic analyses have placed *Argonauta argo* together with their congeners (e.g., *Argonauta hians*), forming a monophyletic Argonautidae, which then forms a sister relationship with the blanket octopuses (e.g., *Tremoctopus*), further forming the superfamily Argonautoidea (Strugnell et al. 2006; Sanchez et al. 2018; Hirota et al. 2021). Although the consensus phylogeny also suggested that Argonautoidea split from benthic ancestral octopods, members of the superfamily including *Arg. argo* are fully adapted to the holopelagic lifestyle and thus do not require any contact with the seafloor throughout its lifecycle. Several studies have suggested that the holopelagic lifestyle was probably achieved through evolutionary acquisitions of distinct characters, enabling members of Argonautoidea to keep afloat in midwater and to brood on their eggs away from the sea floor (cf. Naef 1923; Young 1985; Packard and Wurtz 1994; Bizikov 2004). Buoyancy in argonauts was probably obtained after their ancestors had already become pelagic, potentially via the pelagic paralarval or juvenile stages found in many benthic octopuses with small eggs (Finn and Norman 2010).

One conspicuous characteristic separating Argonautidae, a family that includes all argonauts (genus *Argonauta*), with the rest of Argonautoidea is the presence of a biomineralized eggcase in females, whose external morphology mimics the spirally wound shells of *Nautilus* and the extinct ammonites (Scales 2015; Stevens et al. 2015). The eggcase is thought to protect the eggs laid inside, as well as taking in air to maintain buoyancy (Finn and Norman 2010). As such, the reacquisition of this shell-like structure was probably important because it helps *Argonauta* to maintain its holopelagic lifestyle. Previous observations have maintained that the “shell-like” eggcase is not a “true” shell (the Conchiferan shell; Naef 1923).

The evolutionary story of shell formation and loss in cephalopods is interesting. They have been classified as Conchifera, a subphylum of mollusks composed of members with external shells biomineralized with calcium carbonate. However, except for the nautiloids (Setiamarga et al. 2021a), extant Cephalopods mostly degenerate their shells, resulting in complete shell loss in octopods and vestigial shells in some decapods (squids and cuttlefishes; Kröger et al. 2011). True Conchiferan shells are formed through the secretion of proteins from the mantle tissue, made from aragonite and calcite, have a nacreous layer and intricate microstructures (Jackson et al. 2010, 2017; Kocot et al. 2016), which have evolved since at least the late Ordovician period (Vendrasco et al. 2011, 2013). Within the extant member of the Cephalopods, Nautiloid also forms their shells in this manner (Marie et al. 2009; Setiamarga et al. 2021a).

Despite convergence in their general external morphology, the eggcase of *Argonauta* is not considered as a true Conchiferan shell but as an evolutionary innovation of the genus (Naef 1923; Scales 2015). It is formed through the secretion of related proteins from their arms (Naef 1923; Scales 2015) and has different biomineralization and microstructural profiles (Revelle and Fairbridge 1957; Mitchell et al. 1994; Brain 2005; Saul and Stadum 2005; Oudot et al. 2020). Previously, we conducted an extensive multi-omics analysis of the eggcase of two argonaut species, *Arg. argo* and *Arg. hians*, the samples of which were obtained from the Sea of Japan (Setiamarga et al. 2021b). Two important points relevant to the present study could be taken from the previous study: (1) almost no Conchiferan homologous shell matrix protein (SMP), including those of *Nautilus pompilius* (Setiamarga et al. 2021a), was present in the eggcase matrix of the two argonaut species, and (2) Conchiferan SMP homologs (or homologous domains) were also found in the genome of the shell-less octopods, *Octopus bimaculoides.* These observations indicate that the result is in agreement with those obtained from morphological observations, which indicates that the eggcase is not a homologous structure of the shell. However, the observations raise the question of whether the SMP genes, which are not used in the eggcase formation, are still retained in the genomes of the argonauts. Comparative genome analyses across Cephalopoda, and among different representative species of Conchifera, are required to answer this question. Such genome-level comparative studies would also provide important insights into the evolution of the holopelagic lifestyle at the genetic level.

Until very recently, the lack of genome data has prevented us from understanding the genetic basis of cephalopod biology, and even molluscan biology. This was remedied by recent reports of various cephalopod genomes, such as those of *O. bimaculoides* (Albertin et al. 2015), *Euprymna scolopes* (Belcaid et al. 2019), *Architeuthis dux* (da Fonseca et al. 2020), and *N. pompilius* (Huang et al. 2021; Zhang et al. 2021). Prior this study, although available cephalopod draft genome sequences already covered major extant groups (subclasses Nautiloidea and Coleoidea, and the two major Coleoidea orders, Decabrachia and Octobrachia), genomic data of some key taxa such as Vampyromorpha, amphitretids and the argonauts were not available. The argonaut *Arg. argo*, which is a member of the superfamily Argonautoidea together with the blanket octopus (Tremoctopodidae), forms a sister clade to suborder Incirrata, which includes benthic octopods (Tanner et al. 2017; Sanchez et al. 2018; Hirota et al. 2021). Therefore, the draft genome sequence of this species fills in the gaps of genomic information of the cephalopods, and thus allows discussions to be phylogenetically anchored.

Comparative genomics studies of these genomes have allowed us to identify notable characteristics of cephalopod genomes, except for *Nautilus*, such as: (1) the average genome size of around 3 Gigabases (Gb), which is slightly larger than that of other molluscan species (Gregory 2021), (2) highly rearranged genome with transposable element expansion, which have caused the genomes to be highly repetitive in nature (Albertin et al. 2015; da Fonseca et al. 2020), (3) lineage-specific duplication of certain types of genes (Yoshida et al. 2011), and (4) whole-transcript-wide adenosine to inosine (A-to-I) RNA editing (Alon et al. 2015; Liscovitch-Brauer et al. 2017). These genomic characteristics have thus suggested that coleoid cephalopods have intriguingly different genomes from “standard” metazoan genomes. Another interesting point is that such differences were apparently evolutionarily acquired in ancestral Coleoids, in which members show similar body plans and morphology in general (Young 1971) despite their ancient divergence (Decapodiformes [squid and cuttlefishes] vs. Octopodiformes [vampire squid and octopuses] = 242 ± 38 Ma; Nautiloidea vs. Coleoidea = 415 ± 60 Ma; Kröger et al. 2011; Vinther et al. 2012; Sanchez et al. 2018). Therefore, additional genomic data, especially of the Octopodiformes, will allow us to trace the ancestral chromosomes of Cephalopods and their transition within Mollusks, which might help to unravel the evolutionary origin of these “genomic idiosyncrasies.” These are major obstacles to tracing the ancestral chromosomes of cephalopods and their transition within the Mollusks.

Here, we report a high-quality draft genome assembly of the greater argonaut *Arg. argo*. We found that this species has an exceptionally small genome size, making it an ideal species for genomic studies. Studies targeting argonauts have not progressed because they are difficult to maintain in aquaria; however, we have access to a location in the Sea of Japan from where fresh and living samples of this species could be easily obtained using fixed nets from June to August (Sakurai and Kawano 2010). Using the obtained genome data, we focused on the evolution of some interesting genomic features, such as those related to shell evolution, eggcase formation, and color vision. This allowed us to gain insights into the genomic basis of adaptation to the open-ocean holopelagic lifestyle of this species in particular, and the evolution of cephalopods in general.

## Results and Discussion

### The Draft Genome Assembly of *Arg. argo*

**Fig. 1.**
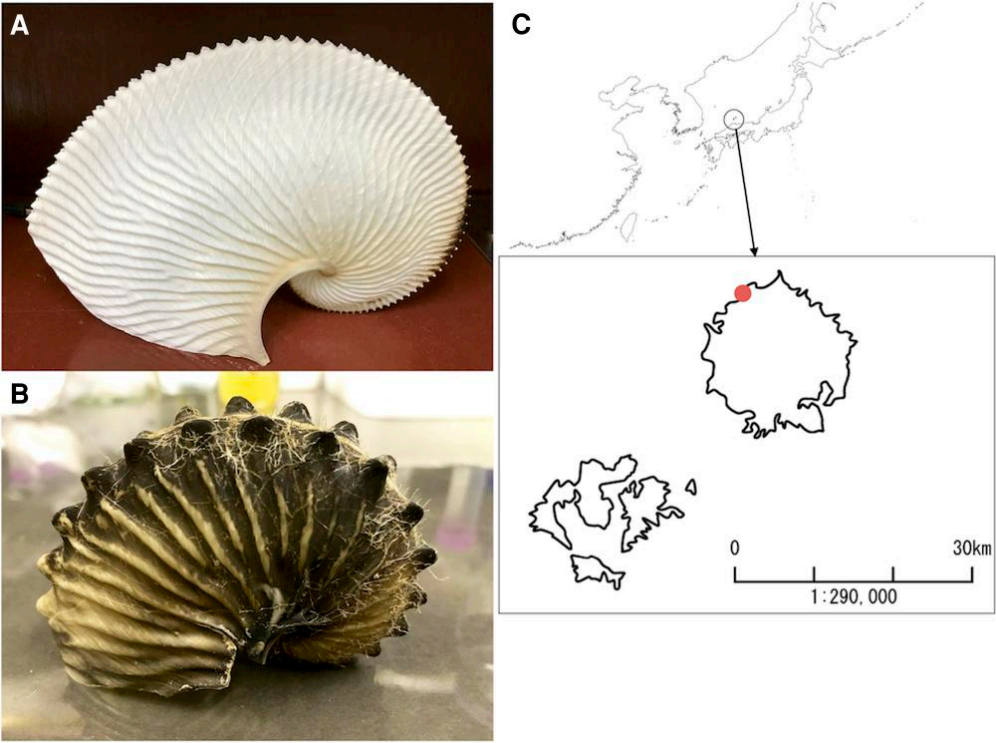
The Argonaut octopuses. (*A*) The shell-like eggcase of *Argonauta argo.* (*B*) The shell-like eggcase of *Argonauta hians.* (*C*) Collect location (map data were created with SimpleMappr, https://www.simplemappr.net).

We generated a draft genome from a single individual of a female argonaut obtained from a fixed net set on the coasts of Oki Island, Shimane Prefecture, Japan (fig. 1). A total of 1.34 Gb was assembled from input sequences obtained using genome sequencing with 201× coverage (107× PE, 24× 3 kb MP, 24× 6 kb MP, 24× 10 kb MP, and 24× 15 kb MP). A total of 57,036 scaffolds of various lengths were assembled, with the top 215 scaffolds larger than 1,000 kb (average length 5,064,479 bp), covering 81% (1.09 Gb) of the total assembly. Half of the assembled scaffolds (N50) were 6.18 Megabases (Mb) or longer, reflecting high contiguity. These statistics (supplementary table S1, Supplementary Material online) thus showed that the *Arg. argo* scaffold sequence ranks among the top-quality draft genomes of Molluscs, and the most comprehensive for Cephalopods. For example, the N50 indicates that this *Arg. argo-draft* genome is twice as long as that of the Hawaiian bobtail squid *E. scolopes* (Belcaid et al. 2019).

The discrepancy between GenomeScope estimation (1.1 Gb, supplementary fig. S1, Supplementary Material online) and the actual assembly size (1.34 Gb; 1.25 Gb non-gap regions) might be caused by the presence of bacterial contamination and/or heterogeneities caused by large insertions and deletions between haploid genomes. However, we only found a minute amount of bacterial genome contamination in this assembly, indicating that the latter was most likely the main cause of the discrepancy. To assess the completeness of the gene space of the assembly, an analysis using BUSCO v3.0.2 (genome mode; Simão et al. 2015) was performed using the provided metazoan data set (metazoa_odb9, *n* = 978), resulting in the recovery of 91.1% of the predicted gene sequences (supplementary table S2, Supplementary Material online). Krait analysis showed that the microsatellite regions accounted for 4.6% of the genome, with dimer and trimer regions accounting for >85% of the region (supplementary fig. S2 and table S3, Supplementary Material online).

The high-quality and relatively high level of completeness of the genome assembly provided in this study, as shown by the statistical analysis presented above, will allow us to address some lingering questions on cephalopod biology and evolution at the genetic and genomic levels. For example, future studies might utilize the microsatellite regions, which comprise a part of the repeat regions in the genome, as individual markers because of the large polymorphisms within individuals, or as markers for paternity analysis of egg masses.

### Ancient Gene Clusters in the Cephalopods: HOX, Parahox, and Wnt Genes

**Fig. 2.**
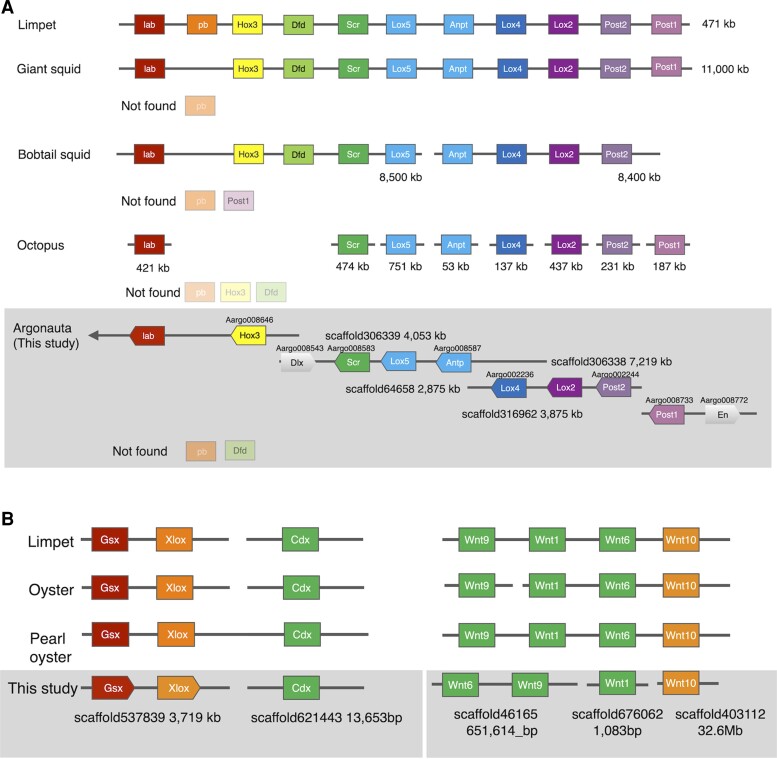
Schematic representations of Hox/Parahox/Wnt clusters. (*A*) Simplified classification of the Hox cluster genomic organization of the cephalopods with the genome sequenced. Scaffold number and length are shown for the *Argonauta argo* genome. The gene model IDs of each gene are shown above each box. The sequences of the homeobox region were confirmed from scaffold for those gene IDs not listed. Hox2/pb and Hox4/Dfd were also not found in the *Arg. argo* genome as in the *Octopus bimaculoides* genome. (*B*) Simplified classification of the Parahox and Wnt cluster genomic organizations of molluscs with the genome sequenced. Scaffold number and length are shown for the *Arg. argo* genome.

The improved contiguity of this genome assembly confirmed the presence of a Hox cluster. The Hox complex, an ancient cluster of transcription factors containing homeodomains, is a famous example of conserved microsynteny (Duboule 2007). The Hox genes of the octopus are atomized (type A: placed independently on the genome; da Fonseca et al. 2020) and thus untypical of the bilaterians (Albertin et al. 2015). When compared with most bilaterians for which draft genome sequences are presently available, the situation is exceptional, and has been the focus of attention because this could be related to major transitions of the metazoan body plan. When we checked it in the *Arg. argo* genome, a large Hox cluster of nine Hox genes on four separate scaffolds was recovered, totaling to a length of at least 18 Mb (fig. 2). Three of the nine Hox genes were not presumed to be gene models, but we used the Homeobox domain sequence to confirm that they are indeed present on the scaffold and that they are Hox genes compared with other Lophotrochozoa genes (supplementary figs. S3 and S4, Supplementary Material online). *Hox2*/*proboscipedia* (*pb*) was not found, as in squid genomes (Belcaid et al. 2019; da Fonseca et al. 2020) except *for Nautilus* (Zhang et al. 2021). We also could not find *Hox4*/*Deformed* (*Dfd*), which is similar to *O. bimaculoides* (Albertin et al. 2015); it is probably a common feature in octopods (supplementary fig. S3, Supplementary Material online). There are at least ten open reading frames (ORFs) inserted among several different Hox genes (three between *Scr* and *Antp*, seven between *Lox4* and *Post2*). Interestingly, no homolog was found in other organisms, including the giant squid *Arc. dux* (da Fonseca et al. 2020), for any of these ORFs (supplementary table S4, Supplementary Material online).

Hox clusters are usually found in contigs of approximately 100 kb in vertebrates and >1,000 kb in invertebrates (Powers et al. 2000; Wagner et al. 2003). Meanwhile, the octopus Hox gene cluster is apparently fragmented, and the genes are present separately on its genome (Albertin et al. 2015), unlike most other bilaterian genomes (Duboule 2007). Intuitively, this finding seems to be in accordance with the staggered, non-colinear expression pattern of Hox genes in cephalopods (Lee et al. 2003; Wollesen et al. 2018). However, the discovery of a Hox cluster in the genome of *Arg. argo*, albeit incomplete, suggests that fragmentation of the cluster is probably a feature limited to benthic octopods (or even a possible artifact of the genome assembly process of *O. bimaculoides*).

The presence of ORFs among several Hox genes in the genome of *Arg. argo* is also intriguing, since it might indicate that the Hox cluster is actually breaking at the location where the intervening genes are located. The Patellogastropod limpet *Lottia gigantea*, another member of the shelled mollusks (Conchifera), was found to have a typical invertebrate Hox cluster spanning 471 kb with no intervening ORFs among any of its Hox genes (Simakov et al. 2013). Recent findings indicate that although the genome of the *N. pompilius* contains a complete set of the molluscan Hox genes, they are not located in a cluster, but are divided into seven contigs (Zhang et al. 2021). On the other hand, the Hox genes in another Cephalopod, the giant squid *Arc. dux*, are present as a disorganized cluster with insertions of intervening non-Hox genes among cluster members (da Fonseca et al. 2020). However, we found no apparent homology or synteny between any of the intervening ORFs of *Arg. argo* and those of *Arc. dux* (supplementary table S4, Supplementary Material online). The acquisition of putative ORFs inside the Hox cluster of *Arg. argo* is probably an indication of a situation that is not dissimilar to that proposed for the fruitfly *Drosophila melanogaster*, in which the Hox cluster is split into two complexes, with non-homeotic genes in between (von Allmen et al. 1996; Wagner et al. 2003; Robertson and Mahaffey 2005), although *Drosophila* still maintained its collinear expression pattern (Graham et al. 1989; Gaunt 2015). However, the Hox cluster break in *Drosophila* is most likely a lineage-specific feature, since the genome of the beetle *Tribolium castaneum* is intact (von Allmen et al. 1996; Shippy et al. 2008; Tribolium Genome Sequencing Consortium 2008). Taken together, it seems that the splits of the Hox cluster could be a symplesiomorphic feature of the Cephalopod genome, but with the actual “Hox de-clustering” processes occurring lineage specifically. This might explain why Cephalopods do not exhibit the typical invertebrate Hox cluster arrangement seen in, for example, the limpet *L. gigantea*.

We also focused on other body plan-related gene microsyntenies (e.g., WNT, Parahox) which remain intact in some bilaterian genomes. The extended Hox complex (*Hox* genes plus *Evx*, *Mox*, and possibly *Dlx*) is also a common feature in bilaterian genomes (Montavon 2015). In vertebrate genomes, the Hox complex has been shown to be linked to the EHGbox (*En*, *Hb9*, and *Gbx*) and NKL gene groups (*Msx*, *Emx*, etc.) and form a supercluster (Garcia-Fernàndez 2005). In the *Arg. argo* genome, we found, probably for the first time in Spiralia, a linkage among *Dlx*, *Engrailed* (*En*), and the Hox genes (fig. 2*A*). In the genome of the giant squid *Arc. dux*, *Dlx* and *En* were found in different scaffolds with no linkage to the Hox cluster (supplementary table S4, Supplementary Material online). However, in *Arg. argo*, *Dlx* was found to be located anterior to Scr relative to its position to the Hox genes (Hox cognate group 4), while *En* was found to be located posterior to *Post1* (fig. 2*A*), thus reversing the presumed ancestral state (Garcia-Fernàndez 2005). Although the possibility of their reinsertion into the Hox group cannot be ruled out, this may indicate that the presence of the extended Hox group is probably conserved in modern cephalopods, although the constraint to preserve gene order is probably relatively weak. The weak constraint in preserving gene order could also explain the “Hox de-clustering,” which includes insertions of ORFs in intergenic regions, observed in cephalopods.

We also observed the presence of the ParaHox cluster, an evolutionary sister complex of the Hox cluster, in the genome of *Arg. argo* (fig. 2*B*). The ParaHox cluster, which consists of the *Gsx*, *Xlox*, and *Cdx* gene families, is a transcription factor involved in anterior–posterior development during early embryogenesis in bilaterians (Brooke et al. 1998; Garstang and Ferrier 2013). The ParaHox cluster is usually found intact in the genomes of Deuterostomes, except for sea urchin and Ascidians (Garstang and Ferrier 2013). However, in Lophotrochozoans, such as the annelid *Platynereis dumerilii* and the limpet *L. gigantea*, only *Gsx* and *Xlox* are clustered together, with *Cdx* broken off and thus unlinked in the genome. The ParaHox cluster of *Arg. argo* was found to conserve the structure of a typical Lophothrocozoan cluster, similar to those reported in *Nautilus* (Huang et al. 2021) and the octopus (Li et al. 2020). Although further studies are still needed, the highly conserved nature of the ParaHox clusters among Cephalopoda, mollusks, and even Lophotrochozoans, indicates the possible presence of an evolutionary constraint to conserve the presence and arrangement of the cluster in the genome, after the breakage of *Cdx* from *Gsx* and *Xlox*.

Similarly, Wnt is an older gene cluster that is widespread in the animal kingdom. Most bilaterian genomes have a common cluster, *wnt9-wnt1-wnt6-wnt10*, or parts of this cluster (Huang et al. 2021). This ancestral cluster of *wnt*s is thought to originate from the evolution of the common ancestor of cnidarians and bilaterians (Janssen et al. 2010; Holstein 2012). In other shelled mollusks (i.e., Conchifera), such as the rock oyster *Crassostrea gigas* and the Japanese pearl oyster *Pinctada fucata*, the limpet *L. gigantea*, and also in *O. bimaculoides*, the *wnt1-wnt6-wnt10* cluster was conserved (Du et al. 2018a), with *L. gigantea* and *Pi. fucata* seemingly retaining some of the lophotrochozoan/protostome *wnt* paralogs (Cho et al. 2010; Setiamarga et al. 2013). In this study, we also confirmed the linkage of *wnt6-wnt9* in *Arg. argo* (fig. 2*B*), in addition to the standard Conchiferan cluster. This suggests that *Arg. argo* probably also derived this arrangement from the ancestral metazoan form of Wnt gene orientation and clustering (*wnt9-wnt1-wnt6-wnt10*). Meanwhile, we also observed a lack of *wnt3* and *wnt8*, which seem to be lost in the ancestral protostomes/lophotrochozoans and in ancestral Conchiferans, respectively (Janssen et al. 2010; Setiamarga et al. 2013; Liu et al. 2018; Bai et al. 2020; Wang et al. 2021).

### Tandem Gene Duplications of Gene Clusters Related to the Pelagic Lifestyle

**Fig. 3.**
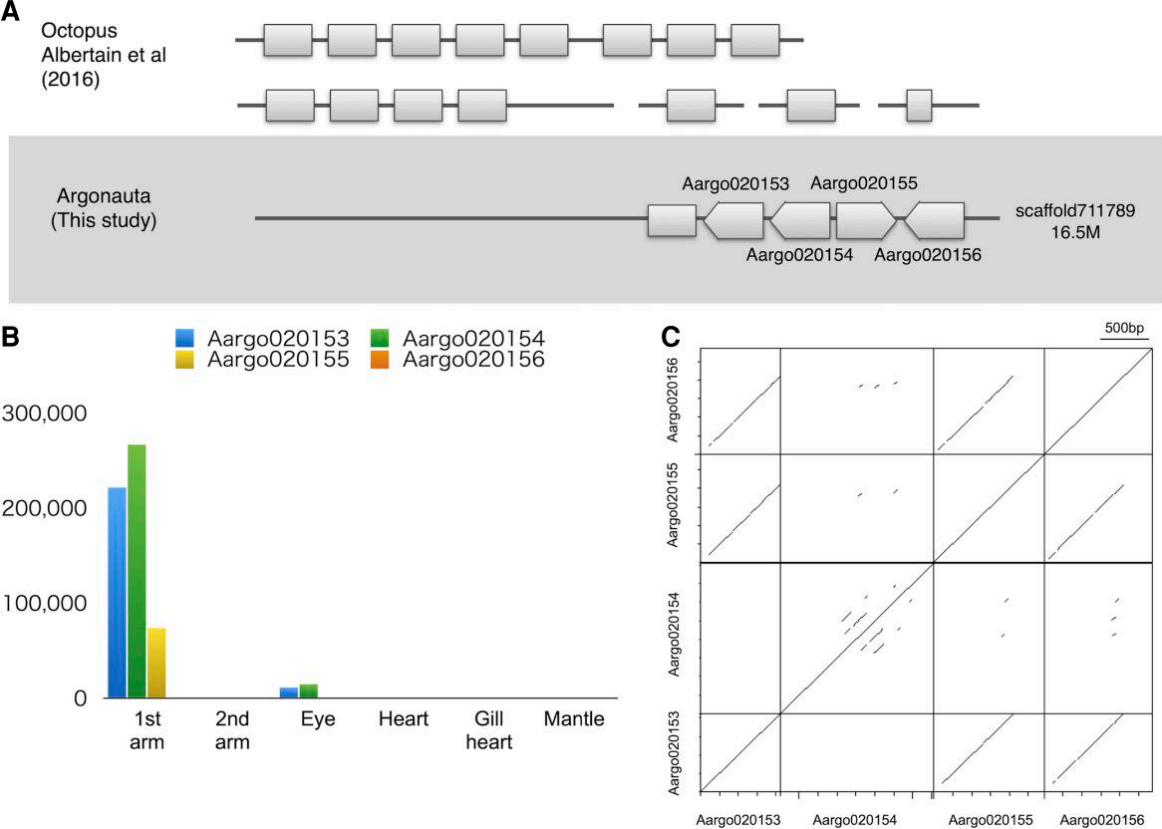
Schematic representations of reflectin clusters. (*A*) Reflectin clusters of the octopuses. (*B*) Gene expression levels of *Argonauta argo* reflectins. (*C*) A DNA dot plot showing the *shared nucleotide region of the four tandemly arranged reflectin CDS sequences*.

**Fig. 4.**
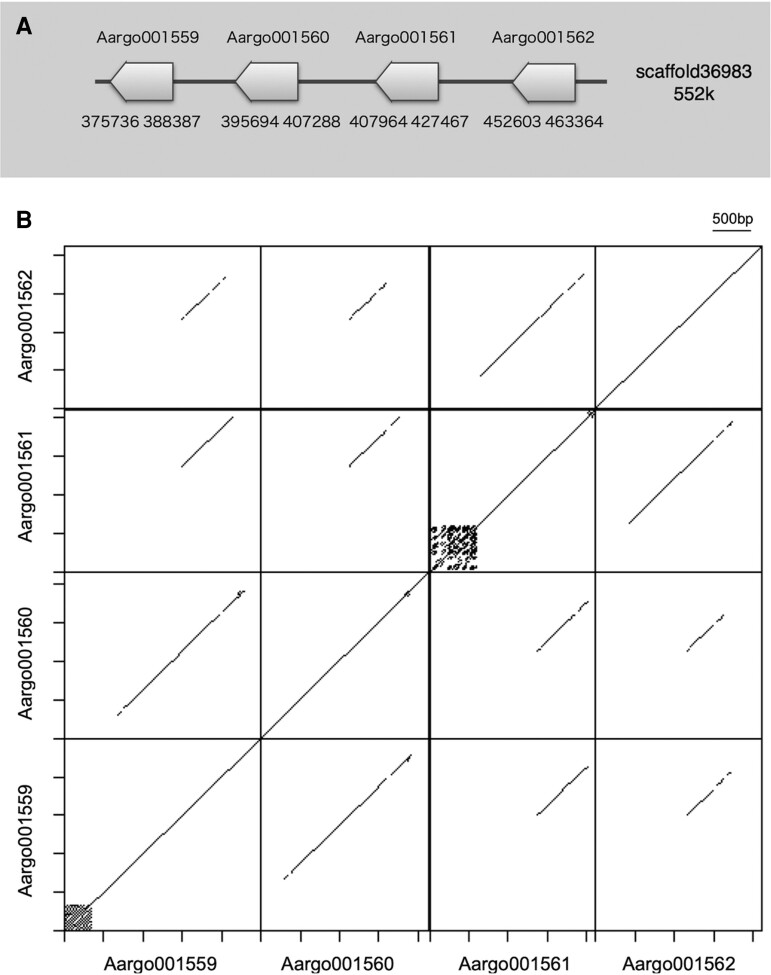
Schematic representations of *tyrosinase* clusters. (*A*) *Tyrosinase* cluster of the argonaut octopus. (*B*) A DNA dot plot showing shared nucleotide regions at the rear of four tyrosinase CDS sequences arranged in tandem.

The *Arg. argo-genome* assembly presented in this study, which is of sufficiently better quality than those of previous octopods, allowed us to investigate the existence of tandem gene arrangements. The searches identified two gene clusters, Reflectins and Tyrosinases (figs. 3 and 4). These genes are highly expressed in the first arms of *Arg. argo* and were suggested to be involved in camouflage and shell formation, which are deemed as important characteristics for adaptation to life in the open sea (fig. 3*B*). Multiple *reflectin* genes have also been identified in other octopuses, and to test whether this was caused by duplication, we checked their location in the genome and the duplication patterns (fig. 3).

Four tandemly arranged gene models of the octopus *reflectin*/*tbc1* domain family (Aargo020153-6) and one possible ORF recovered by a BLAST search in a single scaffold were found in the *Arg. argo* genome (fig. 3*A*). Phylogenetic analysis showed that the three gene models were monophyletic in *Arg. argo* and formed a monophyletic clade together with the sequences of *E. scolopes*, which also formed a monophyletic clade (supplementary fig. S5, Supplementary Material online). The translated sequences of three of the four gene models have at least five of the so-called “Reflectin motifs” (M/FD(X)_5_MD(X)_5_MDX_3/4_) (Levenson et al. 2017, supplementary fig. S6, Supplementary Material online). With only 23 nucleotide substitutions, regardless of codon positions, the coding DNA sequence (CDS) of the tandemly duplicated *reflectin* genes in *Arg. argo* match each other at 97%, covering 760 bp (supplementary fig. S7, Supplementary Material online). Shared nucleotide sequences among the four clustered reflectins were visualized using pairwise dot DNA plot and multiple DNA alignments (fig. 3*C*, supplementary fig. S7, Supplementary Material online). Shared sequence extended >500 nucleotides corresponding to repetitive on the C-terminus end, which correspond to the Reflectin motif of the coding sequence (fig. 3*C*). The sequence similarity was also tested on the genome scaffold coding the reflectins by DNA dot plot analysis (supplementary fig. S8, Supplementary Material online). This plot shows that the four clustered genes share a short repetitive region, which probably correspond to the Reflectin motif on the single exon genes. The high degree of sequence conservation in the reflectins can be attributed to the fact that the motif sequences have a nearly identical sequence. Meanwhile, it is also suggested that the only gene model with a different sequence, Aargo020154, was inverted to the rest of the genes (fig. 3*A*).

There are two possible explanations for the duplicated genes being conserved to form gene clusters: either high level expression is favored and thus retaining duplicated genes would help to increase transcript number, or the multiple copies are conserved under different selection pressures as a result of subfunctionalization (Lynch and Force 2000; Hahn 2009; Morel et al. 2015; Hallin and Landry 2019; Song et al. 2020; Ascencio et al. 2021). It has also been pointed out that the duration of concerted evolution can be influenced by selection for a certain dosage of a gene product; a gene conversion leading to highly similar sequence retention can be advantageous when there is a selection for higher expression levels of that particular gene product, or disadvantageous when divergent gene duplicates are advantageous (Sugino and Innan 2006). Transcriptome analysis showed that in *Arg. argo*, *reflectin* is highly expressed in the first arm and eye, and it seems to be transcribed by the three genes (fig. 3*B*). Therefore, this could support the hypothesis that the cause of gene retention was a high level of expression. This concerted evolution may also be the reason Cephalopod reflectin formed monophyletic clades with members of the clusters within each species (supplementary fig. S5, Supplementary Material online).

The highly expressed *reflectin* is found only in Cephalopods, and the function of the protein products were shown to be related to camouflage by reflecting and refracting light in the surrounding environment (DeMartini et al. 2015). Expressed proteins fill the lamellae of intracellular Bragg reflectors, allowing individuals to exhibit dynamic iris and structural color changes (Crookes et al. 2004). Several tandemly arranged *reflectin* gene clusters have been found in the genome of *E. scolopes*, with the dominant *reflectin* transcripts being almost exclusively expressed in the light organ, eyes, and skin, and thus probably consistent with the development of symbiotic fluorescent organs specifically evolved in this lineage (Belcaid et al. 2019). However, although in *E. scolopes*, the symbiotic luminous organs are important for countershading and survival, no such organ has been found in any of the argonauts. As a defense mechanism, pelagic cephalopods blend into their surroundings by camouflaging, which are done either through translucence or cryptic coloration. The first arm membranes of the argonauts are always wrapped around the shell and reflect light using iridescent chromatophores, causing it to look like a mirror. Meanwhile, the giant squid *Arc. dux* has seven *reflectin* genes and three *reflectin-like* genes in its genome, all except one of which are clustered on the same scaffold (da Fonseca et al. 2020). This non-luminescent deep-sea species has a mirror-like light-reflecting skin for cryptic coloration. These observations indicate that the abundantly expressed Reflectin might help the animals to have light-reflecting mirror-like surfaces, which might then play a role in the ability of these species to blend into their surroundings in the open ocean.

A similar pattern of possible gene conversion was observed in the *tyrosinase* gene cluster. Of the nine *tyrosinase* gene models predicted in *the Arg. argo* genome, eight were of the extracellular or secreted (alpha) type, of which four (Aargo001559-62) were found to be tandemly arranged in a single scaffold (fig. 4). Of the four gene models, excluding unaligned regions, similarities of amino acid sequences of the first two (Aargo001559-60) and the last two (Aargo001561-62) were very high, but there were only 75% similarities between the two gene pairs. These two pairs of tyrosinases are orthologous to closely related molluscan taxa, including the octopus, and form monophyletic groups (supplementary fig. S9, Supplementary Material online). However, the four genes shared an almost exact match in a region in the second half of the gene, at around the 520th–680th aa (fig. 4*B*, supplementary fig. S10, Supplementary Material online). The CDS match rate for this region was 97% with only ca. 60 substitutions, regardless of codon position (supplementary fig. S11, Supplementary Material online). We also constructed a DNA dot plot on the genome scaffold coding the tyrosinases. The dot plot of self-comparison of a 110-kb genomic segment showed that the four tightly clustered *tyrosinase* genes shared sequence extended >1,000 nucleotides on the center of genes (supplementary fig. S12, Supplementary Material online). Since the *tyrosinases* here are a multi-exon gene (4–13 exons were estimated), this suggests that the four *tyrosinase* copies probably underwent gene conversions in two pairs (between Aargo001559 and 1560, between Aargo001661 and 1662) with some partial recombination among the four gene bodies. Gene expression analysis using Stringtie showed that the four have a common gene expression profile, with high levels of expression in the arms and mantle. The phylogenetic tree also indicates that the two pairs of *tyrosinase* genes are apparently orthologous to those found as shell matrix protein-coding genes in Conchiferan mollusks. This finding, that is, the genes expressed only in the arms belong to different gene clusters than those of other tyrosinase-coding genes, might indicate that novel gene paralogs originating from previously existing endogenous *tyrosinase* genes were duplicated and were highly expressed in the arms, possibly used for the calcified eggcase formation, which helps *Arg. argo* and other argonaut octopods to attain buoyancy and thus adapt to their pelagic lifestyle.

### The Evolution of Shell and Eggcase Matrix Proteins Through Independent Recruitments, Losses, and Domain Changes Allows *Arg. argo* to Obtain its Eggcase and Thus its Pelagic Lifestyle

In this study, we found all the eggcase matrix protein-coding genes in the genome of *Arg. argo* (supplementary table S5, Supplementary Material online), as identified by a previous multi-omics study by the authors to survey and identify major proteins of the eggcase matrices of two congeneric argonaut octopods, *Arg. argo* and *Arg. hians* (Setiamarga et al. 2021b). Consistent with previous results, most of the proteins are apparently not shared with the shell matrix proteins of Conchiferans, including those of *Nautilus* (Huang et al. 2021; Setiamarga et al. 2021a), although the genes/proteins themselves are present in the genomes of the Conchiferan mollusks such as the limpet *L. gigantea* (Simakov et al. 2013), the true oyster *C. gigas* (Peñaloza et al. 2021), and the Japanese pearl oyster *Pi. fucata* (Takeuchi et al. 2016). The shell matrix protein-coding genes were also mostly found in the genomes of *Arg. argo* and *O. bimaculoides* (Albertin et al. 2015), indicating their retention despite shell loss in the octopod lineage. Interestingly, the genes for eggcase matrix proteins were also found in the genome of *O. bimaculoides*, and thus, when considered together, supported the previously suggested hypothesis that the argonaut octopods recruited many proteins unrelated to the shell formation and used them for their eggcase (Setiamarga et al. 2021b). Interestingly, some proteins related to calcification, such as Pif-like LamG3, seemed to be used by *Arg. hians* (Setiamarga et al. 2021b).

**fig. 5.**
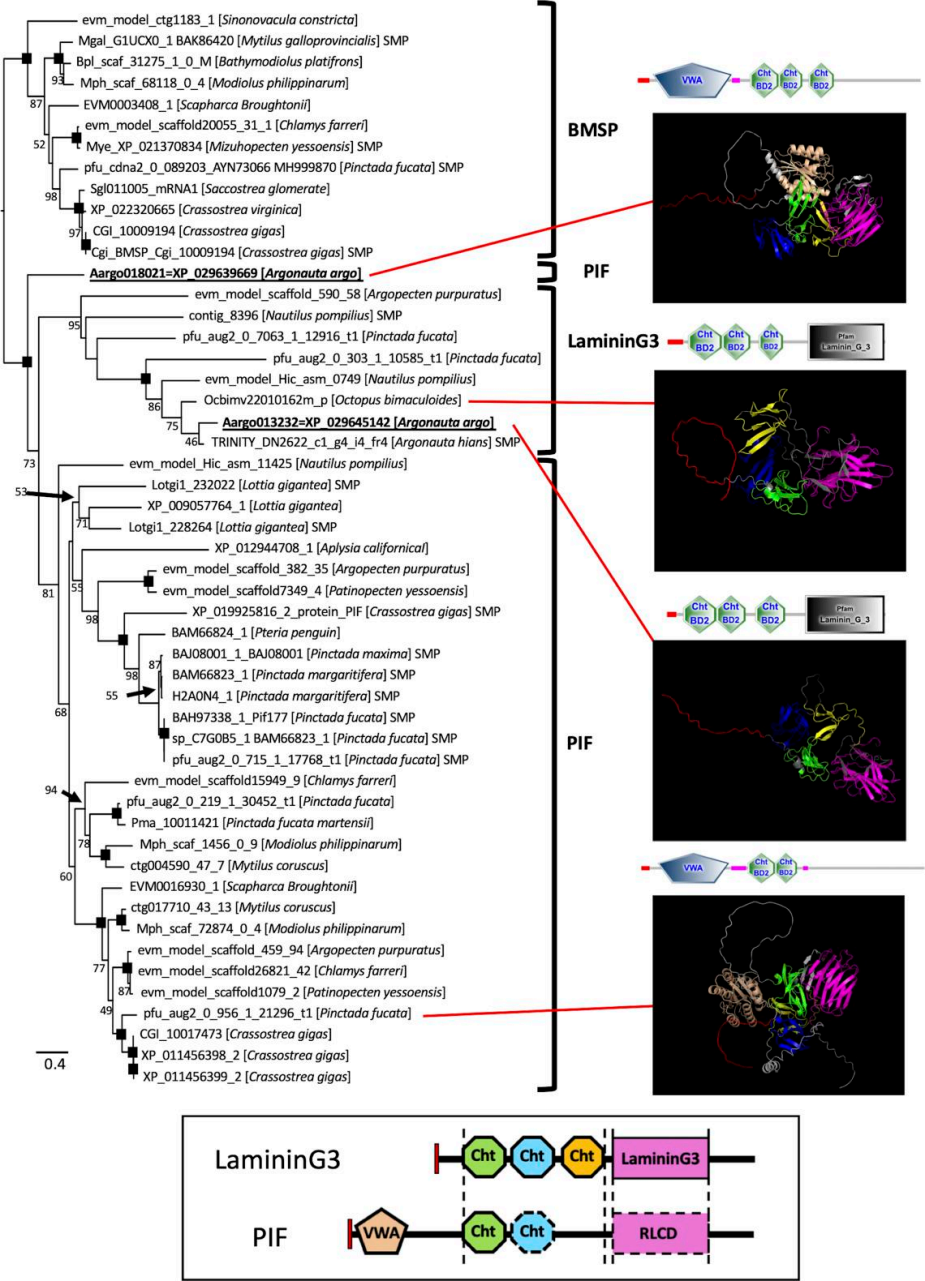
Phylogenetic relationships of Pif/Pif-like/BMSPs of Molluscs and representative 3D protein models. The maximum likelihood tree was estimated under the best fit models (WAG + Γ). Numbers on the nodes are Bootstrap Support (BS) values. BS lower than 41% are shown as “–”, while 100% support is not written. Representative structures of the proteins of the sequences included in the analyses, shown as SMART protein domains, are shown below the trees. Four 3D structural models for PIF (Aargo018021 and one from *Pi. fucata*) and LamG3; (Aargo013232 and Ocbimv22010162m) were estimated with ColabFold v1.0 (AlphaFold2 on Google Colaboratory, Jumper et al. 2021). Schematic representation of domain structure and 3D structural model was colored each domain characteristic according to the color coding in BOX.

In a previous study, we arbitrarily categorized the Pif-like proteins mostly identified as Conchiferan shell matrix proteins into three paralogous groups, based on three monophyletic clades recovered in the phylogeny (see fig. 5 in Setiamarga et al. 2021b), which were also recovered in this study (fig. 5). We arbitrarily named them blue mussel shell protein (BMSP), laminin G3 (LamG3), and Pif, and called them the BMSP/LamG3/Pif proteins. These proteins can be distinguished by their domain combinations. BMSP was first identified as an MP in the blue mussels *Mytilus galloprovincialis* and *L. gigantea* (Suzuki et al. 2011; Marie et al. 2017). The protein has one chitin-binding (ChtBd) and multiple (three or four) von Willebrand factor type A (VWA) domains. BMSP is present throughout the nacreous layer with dense localization in the myostracum, suggesting its possible role in Conchiferan nacreous layer formation (Suzuki et al. 2011). Meanwhile, Pif proteins, which were originally found in the nacre of *Pi. fucata*, usually have two types of domains, one VWA and two ChtBd domains, but with different domain compositions and arrangements (Suzuki et al. 2009; Setiamarga et al. 2021a, 2021b). In vitro functional analysis has shown that it is involved in calcium crystallization (Suzuki et al. 2013).

We did not detect any of the homologs of Pif and BMSP in the eggcase matrix of the argonauts in the previous eggcase study (Setiamarga et al. 2021b), nor in the shell matrix protein study of *Nautilus* (Setiamarga et al. 2021a). LamG3 was first identified by Marie et al. (2017) as one of the two Pif-like isoforms composed of one VWA, three ChtBd, and one LamG domain. In both cephalopods, we found the last type of Pif homologs (*sensu*Setiamarga et al. 2021b), the LamG3 protein, in both the eggcase matrix of *Arg. hians* (but not in the egg matrix proteome and transcriptome of *Arg. argo*), and the shell matrix of *Nautilus*. However, we observed the presence of *lamG3* in the genome of *Arg. argo* (Aargo013232) in this study (fig. 5). Further studies must thus be conducted to assess whether the absence of any transcript/protein product of *lamG3* in *Arg. argo*, despite its presence in the genome, is an artifact caused by the possible non-exhaustiveness of the previous multi-omics study, or if it is not used in the eggcase matrix of *Arg. argo*, which will imply that the eggcases of the two congeneric species are different in nature.

In the previous multi-omics study, we did not find any sequence of *pif* or *BMSP* both in the transcriptome data of all tissues studied and the proteome data of *Arg. argo*; however, we found the presence of an intact coding sequence of *pif* in the genome of the species (Aargo018021, fig. 5). Although the argonaut Pif is not a monophyletic to other molluscan Pif proteins, the domain structure was similar to those of the mollusks (fig. 5). The argonaut Pif does not appear to have any predicted functional domain at the C-terminus, but when we estimated its structure using AlphaFold2, we found that it is very similar to LamG3, suggesting that the two proteins are very close in structure and thus possibly retain the necessary conformation for a shell matrix protein. Further analysis showed that this situation was also true for *Nautilus*. An intact *pif* sequence was also found in the genome of *N. pompilius* (fig. 5, Huang et al. 2021). Unlike LamG3 in the *Nautilus* SMPs, we did not find this *pif* sequence in the transcriptome and proteome data of our recent shell matrix protein multi-omics study of *N. pompilius* (Setiamarga et al. 2021a). The exon–intron structures of each cluster are different and are located at different positions in the genomes of both *Arg. argo* (data not shown) and *Nautilus*. These results seem to indicate that the two Pif homologs (*pif* and *lamG3*) were probably already present separately, at least in the divergence of the nautilus and the extant octopus. The presence of *pif* in the genomes of *Nautilus* and *Arg. argo*, and *lamG3* in the genome of *O. bimaculoides*, even though they are not involved in the formation of shells or shell-like structures, probably because they acquired new functions unrelated to shell formation.

## Conclusion

Until very recently, studies on the evolution of Cephalopoda lacked insights from a genomic perspective. However, recent genome data publications on various species have remedied this. In this study, we presented a genome assembly of *Arg. argo*, which provides significant insight into the genetic and evolutionary background of the adaptation to the pelagic environment, such as the evolution of the visual proteins opsin and reflectin, and the shell matrix protein tyrosinase. The improved quality of the genome assembly also allowed us to identify the presence of regions with high sexual polymorphs, which would be useful in future studies aimed at elucidating the genetic underpinnings of extreme male–female dimorphisms in the species. The pronounced sexual dimorphism probably evolved as an adaptation to holopelagic life in the open ocean with few male–female encounters. In addition, the improved contiguity of the genome assembly confirmed the presence of several gene clusters, including both highly conserved ones, such as Hox, ParaHox, and Wnt, and unique clusters that may be involved in evolutionary novelty.

Previous genome analyses of cephalopods have shown that their genomes are indeed large. The genome of *Doryteuthis* is 1.5 times the size of the human genome, and the *Octopus* genome is 90% of the size of human's. Although there was no evidence for whole-genome duplication occurring in cephalopods, nearly 45% of the assembled genome of the octopus is composed of repetitive elements. The large-sized genomes may allow massive expansions and diversification in several gene families. However, our discovery of species with smaller genome size in the octopus lineage indicates that this trend is not essential for cephalopod evolution. We have also found this exception in another group of squids (Yoshida et al. 2011). The enigma on how the unusual traits of cephalopods, such as their huge brains, could evolve from the molluscan body plan, have attracted the interest of researchers. Therefore, the availability of high-quality genome data of cephalopods with small genome sizes, such as the draft genome of *Arg. argo* reported here, is expected to be a very important source of information for noise-reducing analyses, both for following the evolutionary processes of gene clusters and for future studies of gene regulatory regions.

The newly obtained draft genome of *Arg. argo* also allowed us to hypothesize on the evolution of some major shell matrix proteins related to calcification, seemingly re-recruited in the formation of the eggcase, which was impossible to do in previous multi-omics-based studies. We also corroborated the previous report based on a multi-omics study on eggcase matrix proteins. In this study, we found all of the eggcase matrix proteins previously identified, while also detecting LamG3 in the genome of *Arg. argo* (and *O. bimaculoides*), which was found to be one of the egg matrix proteins of *Arg. hians* but not in *Arg. argo* in the previous multi-omics study. We also found an ortholog of the Pif coding gene in the genome of *Arg. argo*, in addition to the recently published genome of *N. pompilius*. Combined with the protein structure prediction using Alphafold2, we were able to build a hypothesis about how BMSP/LamG3/Pif proteins evolved. We propose that BMSP/LamG3/Pif are key proteins in the formation of calcified external structures, including eggcase. Therefore, the presence of *pif* in the genomes of *Nautilus* and *Arg. argo*, and *lamG3* in the genome of *O. bimaculoides* might explain the usefulness of the LamG3 domain for the formation of calcified structures. This in turn might explain why the argonauts also re-recruited the LamG3 protein, although not necessarily Pif and BMSP, to form their eggs.

## Materials and Methods

### Sampling, Sequencing, and Genome Size Estimation

The *Arg. argo* DNA used for sequencing was derived from a single female caught as a bycatch in the fixed nets set along the coast in Oki Island Town, Shimane Prefecture, Japan (36°17′20.6″ ″N 133°12′46.4″″E). Pieces of the gonad (ovary) were collected from an individual female specimen collected in 2018. The shell is registered as a collection of the University Museum, University of Tokyo, Tokyo, Japan (Voucher No. RM33391). The specimen is also registered under Tree of Life Identifiers; xcArgArgo1. Genomic DNA was extracted from the ovaries using a QIAGEN Genomic-tip kit. Pooled DNA was used for the preparation of three paired-end and three mate-pair (3, 6, 10, and 15 kb insert size) libraries, which were sequenced using an Illumina HiSeq 2500 at the National Institute of Genetics, Japan with support from the Platform for Advanced Genome Science (PAGS; supplementary tables S6–S8, Supplementary Material online).

Pieces of the mantle, arm membrane of the first arm, and second arm tip were obtained from the same single individual to extract genomic DNA. Eyes, hearts, and gill hearts were sampled from different *Arg. argo* individuals. Six transcriptomes of *Arg. argo* were obtained, and raw data statistics are provided in supplementary table S7, Supplementary Material online. Total RNA was extracted from the tissue samples using TRIzol (Invitrogen) followed by an on-column DNase I treatment using the RNeasy mini kit (Qiagen). The RNA was stored at −80 °C until further transcriptome analyses.

The *Arg. argo* genome size and heterozygosity were assessed using GenomeScope v2.0 (Ranallo-Benavidez et al. 2020), based on quality-filtered Illumina reads. A heterozygosity rate of 1.44% was estimated from a 32-mer-based assessment of the *Arg. argo* genome (supplementary fig. S1, Supplementary Material online). Complete microsatellite sequences were estimated and visualized using the Krait v1.3.3 (Du et al. 2018b).

Raw read sequence data are available from the DNA Data Bank of Japan (DDBJ). Genome and transcriptome sequencing reads were deposited in the Sequence Read Archive (Bioproject PRJNA470951). The DDBJ DRA accession numbers are listed in supplementary table S8 (Supplementary material online). To provide these data and information for annotation, we constructed a database, ArgoBase (https://cell-innovation.nig.ac.jp/Aargo/).

### De Novo Genome Assembly and Annotation

Using the predicted 1.1 Gb genome size estimate of *Arg. argo*, the total raw sequence coverage of Illumina reads was 201× (pair-end reads, 3, 6, 10, and 15 kb mate-paired libraries). To reconstruct the mitogenome, we performed contig assembly (-n 200) with Platanus v1.2.4 (Kajitani et al. 2014) using the paired-end data. Contigs annotated as mitochondrial sequences were extracted using the mitogenome data of a closely related species, *Arg. hians* (NC_036354), as the query for BLASTn homology search. After assembling the contigs, both ends of the resulting single contig were manually confirmed to overlap, and redundant parts were removed to complete the full circular mitogenome.

The paired-end sequence reads (PE600) after adapter trimming were assembled using the De Bruijn graph assembler, Platanus-allee v. 2.2.2 (Kajitani et al. 2019). The basic algorithm of Platanus-allee v2.2.2 is based on the arrangement of two independently assembled sequences derived from each haplotype of the corresponding two homologous chromosomes. Contig assembly was performed using only the PE library, and scaffolding and gap closure were performed using all libraries. Assembly statistics for Platanus v222 are shown in supplementary table S9 (Supplementary material online).

Gene prediction models were generated using a custom-made annotation pipeline as previously described (Inoue et al. 2021). In brief, this pipeline combines RNA-seq-based prediction results, homology-based prediction results for related species, and ab initio prediction results using an in-house dynamic program. RNA-seq-based prediction utilized both the assembly first and mapping-first methods. For the assembly first method, RNA-seq data were assembled using Trinity (Grabherr et al. 2011) and Oases (Schulz et al. 2012). The assembled contigs were then splice-mapped using GMAP (Wu and Watanabe 2005). For the mapping-first method, RNA-seq data were mapped to genome scaffolds and the genes were predicted using HISAT2 (Kim et al. 2019) and StringTie (Pertea et al. 2016). For homology-based prediction, the amino acid sequences of *Octopus vulgaris* (Zarrella et al. 2019), *O. bimaculoides* (Albertin et al. 2015), *Arc. dux* (da Fonseca et al. 2020), *C. gigas* (Zhang et al. 2012), and *Mizuhopecten yessoensis* (Wang et al. 2017) were mapped to genome scaffolds using Spaln62, and gene sets were predicted. For ab initio prediction, rain sets were selected from RNA-seq-based prediction results, and AUGUSTUS (Stanke and Waack 2003) and SNAP (Korf 2004) were trained and used for prediction. The predicted results of each tool are shown in supplementary table S10 (Supplementary material online), and as a final result, 20,293 protein-coding genes were predicted (supplementary table S10, Supplementary Material online). The predicted genes were evaluated using BUSCO v3.0.2 (protein mode; Simão et al. 2015) and resulted in 97.0% complete genes being marked, suggesting high accuracy of the annotation (supplementary table S11, Supplementary Material online). This level of accuracy is more than that obtained for cephalopod genomes sequenced so far and is comparable with that of high-quality mollusc genomes (supplementary table S12, Supplementary Material online).

### Phylogenetic Analysis and dot Plot Analysis

Phylogenetic analyses were conducted on five gene families obtained in this study (*Hox*, *reflectin*, *tyrosinase*, *opsin*, and *bmsp/lamg3/pif proteins*). To build single-gene trees based on orthologs, we performed a webBLAST search using *Arg. argo* protein sequences, which were translated from the gene sequences. Sequences for the phylogenetic tree were collected from GenBank to cover the entire Lophotrochozoan clade. To perform multiple alignments of protein sequences, we used the online version of MAFFT v7.487 (Katoh et al. 2002; https://mafft.cbrc.jp/alignment/software/; accessed in August 2021), followed by the removal of ambiguously aligned sites using the online version of trimAlv1.4beta (automated option; Capella-Gutiérrez et al. 2009; http://phylemon2.bioinfo.cipf.es/index.html; accessed in August 2021). The multiple alignment was visualized with Mview (Brown et al. 1998). Maximum likelihood phylogenetic inferences were executed on the software RAxMLGUI v2.0.5 (Silvestro and Michalak 2012; Stamatakis 2006) using the rapid tree search setting with 1,000 bootstrap replications under the best fit models (BMSP/LamG3/Pif proteins = WAG + Γ, Hox = LG + Γ + I, Reflectin = JTT + Γ + F, Tyrosinase = LG + Γ + I). The best-fit models were inferred using the MEGA X (Kumar et al. 2018). The obtained trees were visualized using FigTree v1.4.2 (Rambaut 2010).

Using EMBOSS dotmatcher (Madeira et al. 2022), dot-plot analysis of scaffold sequences encoding tyrosinase and reflectin proteins, respectively, was performed at 200 window size and 50 threshold. The CDS regions were also used for the dot plot analyses using EMBOSS Polydot (Madeira et al. 2022) at default setting. CDS regions were obtained based on the gene annotation obtained above (Argo001559 to Argo001662 for tyrosinases, and Argo020153 to Argo020156 for reflectins).

For opsin, sequences from other metazoans were collected from the GenBank and Ensembl databases. Multiple sequence alignments of the protein sequences were also performed using MAFFT. The best-fit models were inferred using the Modeltest (Darriba et al. 2020). Maximum likelihood phylogenetic inferences were executed on the IQ-TREE software (Minh et al. 2020) with 1,000 bootstrap replications under the best fit models (LG + G4: Best-fit model according to Bayesian Information Criterion (BIC) for c-opsins, LG + F + I + G4: BIC for r-opsins). The trees were visualized using FigTree v1.4.2 (Rambaut 2010).

## Supplementary Material

evac140_Supplementary_Data

## Data Availability

Raw read sequence data are available from the DNA Data Bank of Japan (DDBJ). Genome and transcriptome sequencing reads were deposited in the Sequence Read Archive (Bioproject PRJNA470951). The DDBJ DRA accession numbers are listed in supplementary table S8 (Supplementary material online). A browser of this genome assembly is available at ArgoBase (https://cell-innovation.nig.ac.jp/Aargo/) together with genome scaffolds, gff, and fasta files for gene models (peptides and CDSs).
