# Supplementary material for: Gene Recruitments and Dismissals in the Argonaut Genome Provide Insights into Pelagic Lifestyle Adaptation and Shell-like Eggcase Reacquisition
Source: Genome Biol Evol. 2022 Oct 26;14(11):evac140. doi: 10.1093/gbe/evac140 (PMC9635652; doi:10.1093/gbe/evac140)
Supplement: evac140_Supplementary_Data [file evac140_supplementary_data.pdf]

	Total size (Mbp)	#scaffold	max len (bp)	N50 (bp)	#n50	gap (%)
This study (Platanus v222)	1,342.1	57,036	32,647,630	6,178,209	56	5.30
Octopus vulgaris	2,719.2	13,516	**213,406,131**	**105,892,736**	**10**	**0.03**
Octopus bimaculoides	2,305.8	48,470	4,064,693	485,615	1,300	15.35
Architeuthis dux	2,693.6	7,376	32,889,981	4,852,590	160	16.76
Crassostrea gigas	557.4	**6,433**	1,964,558	402,213	399	11.82
Mizuhopecten yessoensis	971.6	29,088	7,498,238	827,226	334	8.23

Table S1 Assembly comparison among molluscan genomes

BUSCO (%)	complete	single	duplicate	fragmented	missing
This study (Platanus v222)	**91.10**	88.55	2.56	**3.07**	**5.83**
Octopus vulgaris	82.62	80.88	1.74	5.01	12.37
Octopus bimaculoides	89.98	**89.47**	**0.51**	3.68	6.34
Architeuthis dux	88.55	87.63	0.92	3.58	7.87

Table S2 Assembly comparison based on BUSCO scores (genome mode, metazoa_odb9, n=978)

Type	*Counts*	*Length* (bp)	*Percent* (%)	*Average Length* (bp)	*Relative Abundance* (loci/Mb)
Mono	32,911	444,137	1.31	13.5	25.89
Di	1,424,160	40,218,122	56.62	28.24	1,120.52
Tri	729,359	18,793,488	28.99	25.77	573.86
Tetra	301,301	9,745,400	11.98	32.34	237.06
Penta	18,002	433,820	0.72	24.1	14.16

Table S3 Microsatellite sequences estimated by Krait

GeneID	*Aargo scaffold ID*	*Hox gene location*	*Best BLAST hit againt Architeuthis genome*	BLAST hit *start*	*end*	*e-value*	*Identity*
Aargo008646	306339	*Hox3*	VCCN01003880.1	25694950	25695123	1.54e-28	98.3
Aargo008543	306338	*Dlx*	VCCN01000387.1	16435580	16435281	1.27e-54	94.1
Aargo008583	306338	*Scr*	VCCN01003880.1	24018414	24018650	2.20e-45	100
Aargo008584	306338		No hit				
Aargo008585	306338		No hit				
Aargo008586	306338		No hit				
Aargo008587	306338	*Antp*	VCCN01003880.1	22119788	22120258	1.75e-45	74.1
Aargo002236	64658	*Lox4*	VCCN01003880.1	21387779	21388090	2.05e-48	78.4
Aargo002237	64658		No hit				
Aargo002238	64658		No hit				
Aargo002239	64658		No hit				
Aargo002240	64658		No hit				
Aargo002241	64658		No hit				
Aargo002242	64658		No hit				
Aargo002243	64658		No hit				
Aargo002244	64658	*Post2*	VCCN01003880.1	19080283	19080780	4.74e-102	94.0
Aargo008733	316962	*Post1*	VCCN01003880.1	17446089	17445817	1.24e-48	94.5
Aargo008772	316962	*En*	VCCN01004895.1	231027	231302	1.35e-46	89.1

Table S4 Comparison of ORFs in the Hox cluster between the argonaut and giant squid

Accession	Soluble/InSoluble	Score (Soluble)	Score (inSoluble)	Mass	emPAI (Soluble)	emPAI (inSoluble)	Top hit description
Aargo014584	Soluble/InSoluble	1265	1445	18699	5.05	5.05	XP_029645183.1(uncharacterized protein LOC115219204)
Aargo010955	Soluble/InSoluble	3267	1685	70815	3.76	2.1	XP_029648017.1(glypican-6-like)
Aargo013239	Soluble/InSoluble	2618	4552	29045	3.16	6.95	XP_029645506.1(uncharacterized protein LOC115219476)
Aargo009090	Soluble/InSoluble	1863	5981	46847	1.25	2.11	XP_029635552.1(kielin/chordin-like protein)
Aargo007096	Soluble	863		67927	0.66		XP_029641298.1(kielin/chordin-like protein)
Aargo013011	Soluble/InSoluble	1088	822	15665	0.61	0.61	no hit
Aargo015879	Soluble/InSoluble	348	395	18416	0.5	1.25	XP_029639174.1(uncharacterized protein LOC115214207)
Aargo018907	Soluble	73	63	9277	0.48	1.18	no hit
Aargo003237	Soluble/InSoluble	39	109	11360	0.38	1.64	XP_029634140.1(histone H4)
Aargo008472	Soluble	43		27061	0.32		XP_029657877.1(matrix metalloproteinase-19-like)
Aargo019511	Soluble	170		56212	0.31		XP_029657579.1(protein disulfide-isomerase-like)
Aargo000068	Soluble	94		77205	0.28		XP_029641298.1(kielin/chordin-like protein)
Aargo009082	Soluble/InSoluble	572	9891	137812	0.21	1.3	XP_029641298.1(kielin/chordin-like protein)
Aargo010041	Soluble/InSoluble	108	43	63890	0.2	0.06	XP_029649226.1(70 kDa neurofilament protein-like)
Aargo017478	Soluble	68		47245	0.17		XP_029655835.1(collagen alpha-1(XII) chain-like)
Aargo003530	Soluble/InSoluble	51	27	23962	0.17	0.17	XP_029652506.1(uncharacterized protein LOC115225721)
Aargo014807	Soluble	38		25623	0.16		XP_029637330.1(peptidyl-prolyl cis-trans isomerase B-like)
Aargo020077	Soluble	45		68646	0.12		XP_029635321.1(glutathione hydrolase 1 proenzyme-like)
Aargo005089	Soluble/InSoluble	42	35	64734	0.12	0.06	XP_029636026.1(nucleobindin-2-like)

Aargo018523	Soluble/InSoluble	34	30	32307	0.12	0.12	XP_029645142.1(uncharacterized protein LOC115219174)
Aargo009979	Soluble/InSoluble	80	92	41993	0.09	0.2	XP_029636148.1(lysosomal aspartic protease-like isoform X2)
Aargo006874	Soluble/InSoluble	58	74	41917	0.09	0.57	XP_029639043.1(actin, cytoplasmic)
Aargo001902	Soluble	54		43454	0.09		XP_029647804.1(lachesin-like)
Aargo019743	Soluble	35		41792	0.09		XP_029643399.1(glyceraldehyde-3-phosphate dehydrogenase-like isoform X1)
Aargo012655	Soluble/InSoluble	70	36	50731	0.08	0.08	XP_029646382.1(probable peptidylglycine alpha-hydroxylating monooxygenase 1)
Aargo005978	Soluble/InSoluble	54	27	101036	0.08	0.04	XP_029653787.1(matrix metalloproteinase-2-like)
Aargo009281	Soluble/InSoluble	50	38	51291	0.08	0.08	XP_029633267.1(elongation factor 1-alpha-like)
Aargo005181	Soluble	32		52353	0.08		XP_029636907.1(carbohydrate sulfotransferase 11-like)
Aargo009733	Soluble	47		60167	0.07		XP_029640025.1(glypican-3-like)
Aargo004396	Soluble/InSoluble	42	22	60268	0.07	0.07	XP_029652967.1(vascular non-inflammatory molecule 3-like)
Aargo009172	Soluble	72		138099	0.06		XP_029635449.1(Golgi apparatus protein 1-like)
Aargo007715	Soluble	128		483884	0.05		no hit
Aargo013134	Soluble	48		75467	0.05		XP_029645082.1(uncharacterized protein LOC115219132)
Aargo007446	Soluble	48		73037	0.05		XP_029644395.1(endoplasmic reticulum chaperone BiP-like)
Aargo003471	Soluble	69		184525	0.04		XP_029656717.1(uncharacterized protein LOC115230720, partial)
Aargo014038	Soluble	48		89243	0.04		XP_029656725.1(BMP-binding endothelial regulator protein-like)
Aargo008197	Soluble/InSoluble	47	28	96408	0.04	0.04	XP_029651355.1(calsyntenin-1-like)
Aargo017333	Soluble/InSoluble	56	33	116015	0.03	0.03	XP_029636473.1(lysosomal alpha-mannosidase-like)

Aargo014037	Soluble	54		249053	0.02		XP_029653605.1(uncharacterized protein LOC115226724)
Aargo009083	InSoluble		3825	60250		1.13	XP_029641298.1(kielin/chordin-like protein)
Aargo003067	InSoluble		41	13411		0.32	XP_029640572.1(histone H2A)
Aargo003084	InSoluble		43	13546		0.31	XP_029641645.1(histone H2B, gonadal)
Aargo003528	InSoluble		28	29098		0.3	XP_029652588.1(endoplasmic reticulum resident protein 44-like)
Aargo002845	InSoluble		22	15376		0.27	XP_029641651.1(histone H3.3)
Aargo000067	InSoluble		70	88525		0.24	XP_029641298.1(kielin/chordin-like protein)
Aargo002647	InSoluble		35	23029		0.18	XP_029643810.1(ras-related protein Rab-30-like)
Aargo013125	InSoluble		29	31859		0.13	XP_029645353.1(uncharacterized protein LOC115219333)
Aargo002562	InSoluble		28	29878		0.13	XP_029643815.1(14-3-3 family protein artA-like)
Aargo011145	InSoluble		25	33590		0.12	XP_029645177.1(arginase-1-like)
Aargo008234	InSoluble		24	48249		0.08	XP_029651017.1(tryptophan--tRNA ligase, cytoplasmic-like)
Aargo002156	InSoluble		23	56509		0.07	XP_029640651.1(ATP synthase subunit beta, mitochondrial)
Aargo001845	InSoluble		75	91175		0.04	no hit
Aargo002777	InSoluble		42	90648		0.04	XP_029653634.1(WD repeat-containing protein on Y chromosome-like)
Aargo002913	InSoluble		25	469668		0.01	XP_029641431.1(spectrin beta chain, non-erythrocytic 1-like isoform X2)

Table S5 The list of EsMPs based on *Argonauta argo* gene models

Library types	Read length	Number of reads	Total nucleotides	Total nucleotide after trimming
PE600	250	235M	117.5Gb	109.0Gb
MP3kb	100	129M	25.9Gb	14.7Gb
MP6kb	100	130M	25.9Gb	16.5Gb
MP10kb	100	129M	25.8Gb	17.0Gb
MP15kb	100	132M	26.4Gb	16.8Gb

Table S6 *A. argo* genome sequencing data

Tissues	Read length	Number of reads
heart	100	26.3M
GH	100	27.3M
eye	100	24.9M
1st	101	26.0M
2nd	101	27.4M
mantle	101	26.8M

Table S7 *A. argo* transcriptome sequencing data

Category	Species	Tissue	BioProject ID	BioSample ID	DRA ID	DRA Accession numbers
A.argo genome	A.argo	Ovary	PRJDB8915	SAMD00191535	DRA009189	DRX187074
						DRR196657
A.argo genome	A.argo	Ovary	PRJDB8915	SAMD00191535	DRA009189	DRX187075
						DRR196658
A.argo genome	A.argo	Ovary	PRJDB8915	SAMD00191535	DRA009189	DRX187076
						DRR196659
A.argo genome	A.argo	Ovary	PRJDB8915	SAMD00191535	DRA009189	DRX187077
						DRR196660
A.argo genome	A.argo	Ovary	PRJDB8915	SAMD00191535	DRA009189	DRX187078
						DRR196661
A.argo RNA	A.argo	heart	PRJDB8915	SAMD00191536	DRA009189	DRX187079
						DRR196662
A.argo RNA	A.argo	GH	PRJDB8915	SAMD00191537	DRA009189	DRX187080
						DRR196663
A.argo RNA	A.argo	eye	PRJDB8915	SAMD00191538	DRA009189	DRX187081
						DRR196664
A.argo RNA	A.argo	1st arm	PRJDB9668	SAMD00216778	DRA010000	DRX212040
						DRR221727
A.argo RNA	A.argo	2nd arm	PRJDB9668	SAMD00216777	DRA010000	DRX212041
						DRR221728
A.argo RNA	A.argo	mantle	PRJDB9668	SAMD00216776	DRA010000	DRX212042
						DRR221729
A.hians RNA	A.hians	1st arm	PRJDB9668	SAMD00216781	DRA010001	DRX212043
						DRR221730
A.hians RNA	A.hians	2nd arm	PRJDB9668	SAMD00216780	DRA010001	DRX212044
						DRR221731
A.hians RNA	A.hians	mantle	PRJDB9668	SAMD00216779	DRA010001	DRX212045
						DRR221732

Table S8 List of accession numbers obtained in this study

	consensus	*phased (primary)*
Number of scaffolds (≧500bp)	57,036	8,338
Total length	1,342,119,551 bp	1,105,831,271 bp
Longest scaffold	32,647,630 bp	13,511,555 bp
N50 (scaffold, ≧500bp)	6,178,209 bp	1,690,686 bp
L50 (#:scaffold, ≧500bp)	56	161
N (bp, %)	71,142,411 bp (5.3%)	59,948,776 bp (5.4%)
Number of contigs (≧500bp)	161,761	90,477
Total length	1,266,747,862 bp	1,044,535,279 bp
Longest contig	443,220 bp	440,694 bp
N50 (contig, ≧500bp)	22,401 bp	24,544 bp
L50 (#:contig, ≧500bp)	14,005	10,963

Table S9 Assembly statistics by *Platanus* v222

Prediction		*#gene*	*exon/gene*	*#single exon genes*	*Average exon length* (bp)	*Average CDS length* (bp)	*Average intron length* (bp)
RNA-seq	mapping	25,952	6.44	7,777	184.16	1,186.45	3,033.56
	de novo	71,352	6.58	21,744	175.18	1,152.55	3,145.65
homology		121,140	11.03	10,643	143.98	1,587.64	3,357.91
ab initio	Augustus	25,470	4.78	7,314	221.76	1,059.19	3,964.98
	SNAP	80,685	4.82	5,798	113.38	546.24	4,073.65
Consensus		20,293	6.9	1,393	181.51	1,252.54	3,038.45

Table S10 Gene prediction models using custom-made annotation pipeline with transcriptomic data (Inoue et al. 2021)

BUSCO (%)	complete	single	duplicate	fragmented	missing
This study (Platanus v222)	**97.03**	93.56	3.48	**1.43**	**1.53**
Octopus vulgaris	97.96	96.42	1.53	0.92	1.12
Octopus bimaculoides	94.79	93.66	**1.12**	2.45	2.76
Architeuthis dux	88.45	87.12	1.33	7.87	3.68

Table S11 Gene model comparison based on BUSCO scores (protein mode, metazoa_odb9, n=978)

	#gene	Number of exons per gene	#single exon genes	Average exon length (bp)	Average CDS length (bp)	Average intron length (bp)	GT-AG splice (%)
This study	20,293	6.90	1,393	181.51	1,252.54	3,038.45	98.01
Octopus vulgaris	18,183	7.93	3,121	262.50	1,542.68	6,376.70	98.46
Octopus bimaculoides	15,842	8.35	1,727	256.69	1,547.02	5,280.46	98.09
Architeuthis dux	33,406	5.08	5,290	200.48	1,015.48	3,400.01	99.23
Crassostrea gigas	28,402	7.91	1,358	272.60	1,483.46	923.43	98.39
Mizuhopecten yessoensis	24,521	8.48	1,891	345.73	1,660.85	2,217.62	98.86

Table S12 Gene model comparison among molluscan genomes

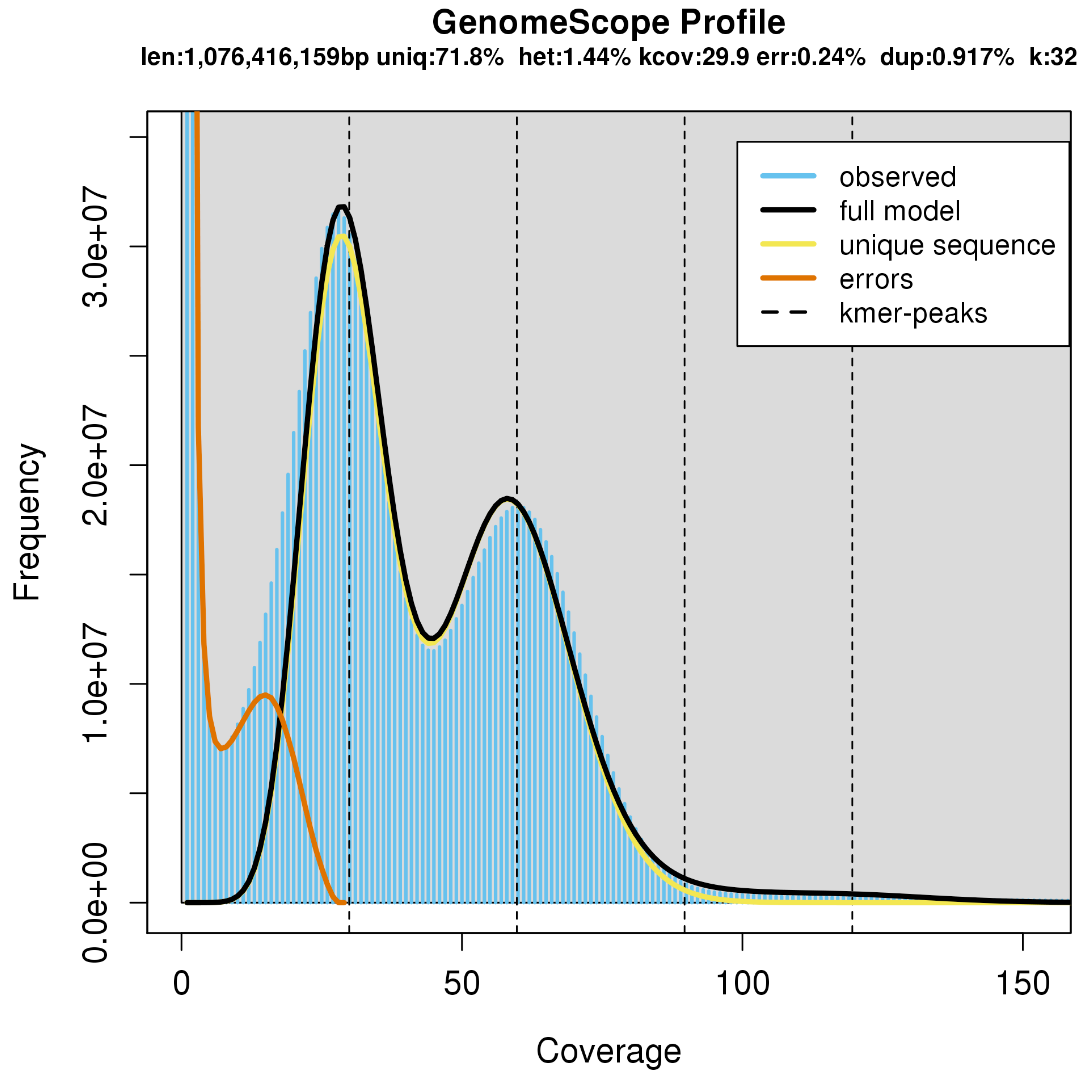

Figure S1 GenomeScope estimation of genome size based on k-mer coverage (k=32).

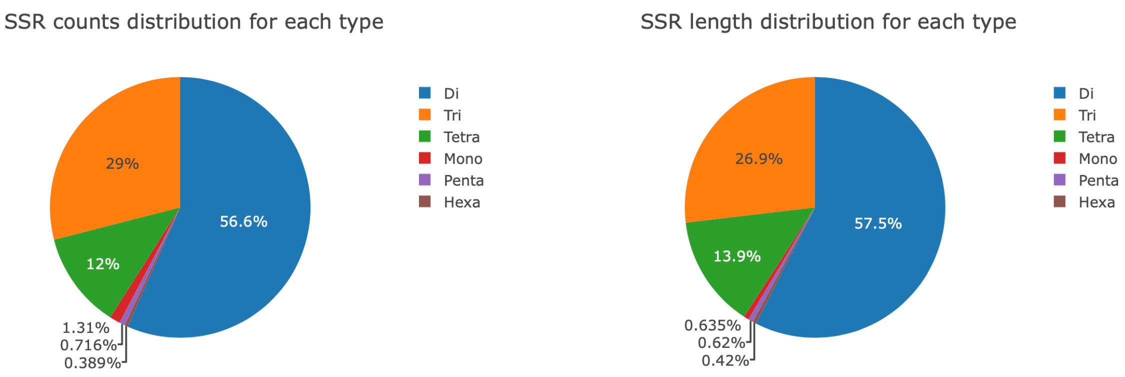

Figure S2 Microsatellite types found in the Argonaut genome.

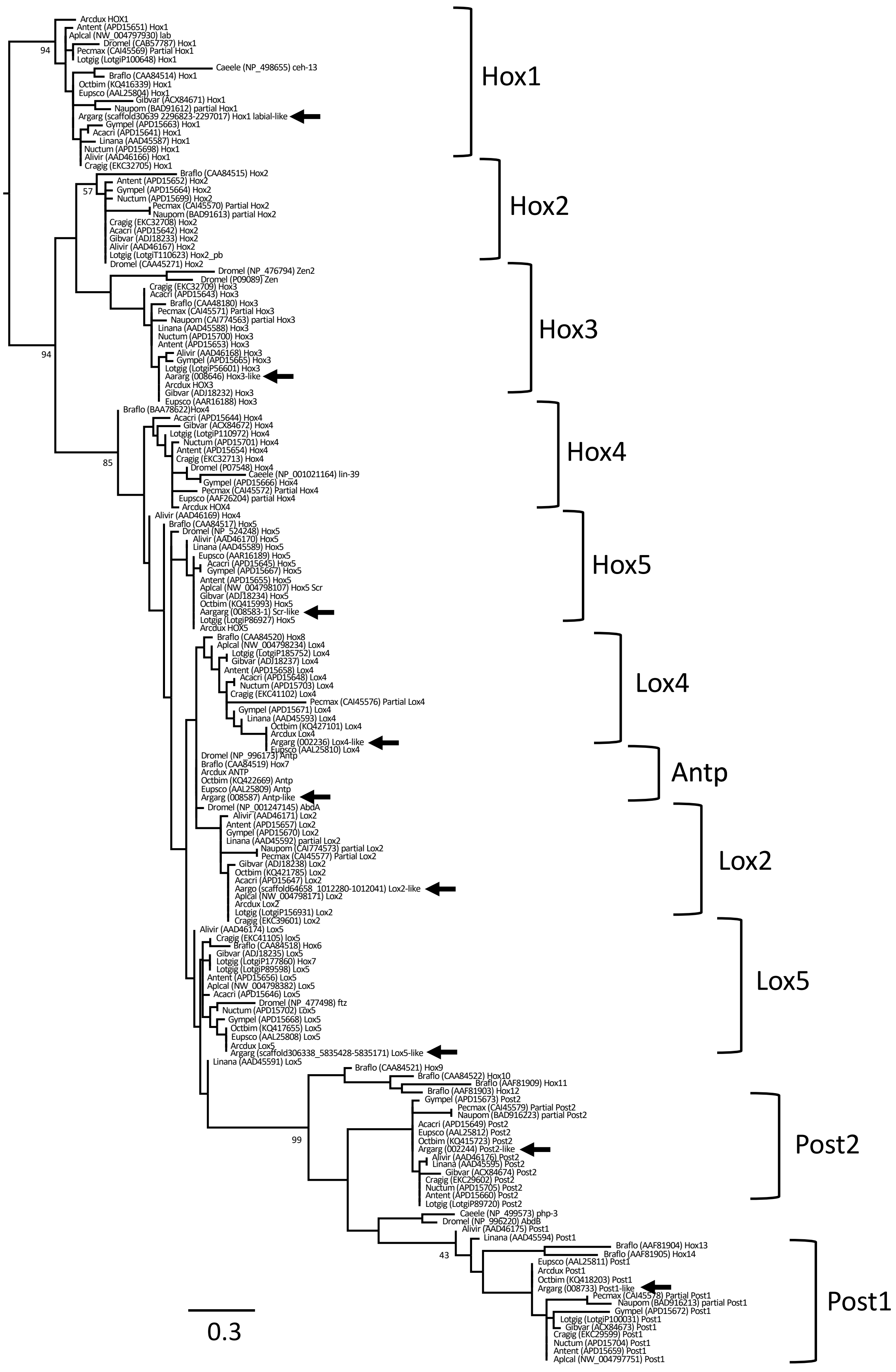

Arcdux HOX1
Antent (APD15651) Hox1
Aplcal (NW_004797930) lab
Dromel (CAB57787) Hox1
Pecmax (CAI45569) Partial Hox1
Lotgig (LotgiP100648) Hox1
Caeele (NP_498655) ceh-13
Braflo (CAA84514) Hox1
Octbim (KQ416339) Hox1
Eupsco (AAL25804) Hox1
Gibvar (ACX84671) Hox1
Naupom (BAD91612) partial Hox1
Argarg (scaffold30639 2296823-2297017) Hox1 labial-like
Gympel (APD15663) Hox1
Acacri (APD15641) Hox1
Linana (AAD45587) Hox1
Nuctum (APD15698) Hox1
Alivir (AAD46166) Hox1
Cragig (EKC32705) Hox1
94
Braflo (CAA84515) Hox2
Antent (APD15652) Hox2
Gympel (APD15664) Hox2
Nuctum (APD15699) Hox2
Pecmax (CAI45570) Partial Hox2
Naupom (BAD91613) partial Hox2
Cragig (EKC32708) Hox2
Acacri (APD15642) Hox2
Gibvar (ADJ18233) Hox2
Alivir (AAD46167) Hox2
Lotgig (LotgiT110623) Hox2_pb
Dromel (CAA45271) Hox2
57
Dromel (NP_476794) Zen2
Dromel (P09089) Zen
Cragig (EKC32709) Hox3
Acacri (APD15643) Hox3
Braflo (CAA48180) Hox3
Pecmax (CAI45571) Partial Hox3
Naupom (CAI774563) partial Hox3
Linana (AAD45588) Hox3
Nuctum (APD15700) Hox3
Antent (APD15653) Hox3
Alivir (AAD46168) Hox3
Gympel (APD15665) Hox3
Lotgig (LotgiP56601) Hox3
Aararg (008646) Hox3-like
Arcdux HOX3
Gibvar (ADJ18232) Hox3
Eupsco (AAR16188) Hox3
94
Braflo (BAA78622)Hox4
Acacri (APD15644) Hox4
Gibvar (ACX84672) Hox4
Lotgig (LotgiP110972) Hox4
Nuctum (APD15701) Hox4
Antent (APD15654) Hox4
Cragig (EKC32713) Hox4
Dromel (P07548) Hox4
Caeele (NP_001021164) lin-39
Gympel (APD15666) Hox4
Pecmax (CAI45572) Partial Hox4
Eupsco (AAF26204) partial Hox4
Arcdux HOX4
85
Alivir (AAD46169) Hox4
Braflo (CAA84517) Hox5
Dromel (NP_524248) Hox5
Alivir (AAD46170) Hox5
Linana (AAD45589) Hox5
Eupsco (AAR16189) Hox5
Acacri (APD15645) Hox5
Gympel (APD15667) Hox5
Antent (APD15655) Hox5
Aplcal (NW_004798107) Hox5 Scr
Gibvar (ADJ18234) Hox5
Octbim (KQ415993) Hox5
Aargarg (008583-1) Scr-like
Lotgig (LotgiP86927) Hox5
Arcdux HOX5
Braflo (CAA84520) Hox8
Aplcal (NW_004798234) Lox4
Lotgig (LotgiP185752) Lox4
Gibvar (ADJ18237) Lox4
Antent (APD15658) Lox4
Acacri (APD15648) Lox4
Nuctum (APD15703) Lox4
Cragig (EKC41102) Lox4
Pecmax (CAI45576) Partial Lox4
Gympel (APD15671) Lox4
Linana (AAD45593) Lox4
Octbim (KQ427101) Lox4
Arcdux Lox4
Argarg (002236) Lox4-like
Eupsco (AAL25810) Lox4
Dromel (NP_996173) Antp
Braflo (CAA84519) Hox7
Arcdux ANTP
Octbim (KQ422669) Antp
Eupsco (AAL25809) Antp
Argarg (008587) Antp-like
Dromel (NP_001247145) AbdA
Alivir (AAD46171) Lox2
Antent (APD15657) Lox2
Gympel (APD15670) Lox2
Linana (AAD45592) partial Lox2
Naupom (CAI774573) partial Lox2
Pecmax (CAI45577) Partial Lox2
Gibvar (ADJ18238) Lox2
Octbim (KQ421785) Lox2
Acacri (APD15647) Lox2
Aargo (scaffold64658_1012280-1012041) Lox2-like
Aplcal (NW_004798171) Lox2
Arcdux Lox2
Lotgig (LotgiP156931) Lox2
Cragig (EKC39601) Lox2
Alivir (AAD46174) Lox5
Cragig (EKC41105) lox5
Braflo (CAA84518) Hox6
Gibvar (ADJ18235) Lox5
Lotgig (LotgiP177860) Hox7
Lotgig (LotgiP89598) Lox5
Antent (APD15656) Lox5
Aplcal (NW_004798382) Lox5
Acacri (APD15646) Lox5
Dromel (NP_477498) ftz
Nuctum (APD15702) Lox5
Gympel (APD15668) Lox5
Octbim (KQ417655) Lox5
Eupsco (AAL25808) Lox5
Arcdux Lox5
Argarg (scaffold306338_5835428-5835171) Lox5-like
Linana (AAD45591) Lox5
Braflo (CAA84521) Hox9
Braflo (CAA84522) Hox10
Braflo (AAF81909) Hox11
Braflo (AAF81903) Hox12
Gympel (APD15673) Post2
Pecmax (CAI45579) Partial Post2
Naupom (BAD916223) partial Post2
Acacri (APD15649) Post2
Eupsco (AAL25812) Post2
Octbim (KQ415723) Post2
Argarg (002244) Post2-like
Alivir (AAD46176) Post2
Linana (AAD45595) Post2
Gibvar (ACX84674) Post2
Cragig (EKC29602) Post2
Nuctum (APD15705) Post2
Antent (APD15660) Post2
Lotgig (LotgiP89720) Post2
99
Caeele (NP_499573) php-3
Dromel (NP_996220) AbdB
Alivir (AAD46175) Post1
Linana (AAD45594) Post1
Braflo (AAF81904) Hox13
Braflo (AAF81905) Hox14
43
Eupsco (AAL25811) Post1
Arcdux Post1
Octbim (KQ418203) Post1
Argarg (008733) Post1-like
Pecmax (CAI45578) Partial Post1
Naupom (BAD916213) partial Post1
Gympel (APD15672) Post1
Lotgig (LotgiP100031) Post1
Gibvar (ACX84673) Post1
Cragig (EKC29599) Post1
Nuctum (APD15704) Post1
Antent (APD15659) Post1
Aplcal (NW_004797751) Post1
Hox1
Hox2
Hox3
Hox4
Hox5
Lox4
Antp
Lox2
Lox5
Post2
Post1
0.3

Figure S3 Molecular phylogenetic tree of the Hox genes. The maximum likelihood phylogenetic tree inferred under the LG + Γ + I model with 1000 bootstrap replicates. Hox genes of *Argonauta argo* are marked with a black arrow. Abbreviations: Nuctum: *Nucula tumidula*, Cragig: *Crassostrea gigas*, Pecmax: *Pecten maximus*, Gibvar: *Gibbula varia*, Lotgig: *Lottia gigantea*, Apcal: *Aplysia californica*, Eupsco: *Euprymna scolopes*, Octbim: *Octopu bimaculoides*, Naupom: *Nautilus pompilius*, Acacri: *Acanthochitona crinite*, Antent: *Antalis entails*, Glympell: *Gymnomenia pellucida*, Alivir: *Alitta virens*, Linana: *Lingula anatine*: Dromel: *Drosophila melanogaster*, Braflo: *Branchiostoma floridae*: Caeele, *Caenorhabditis elegans*.

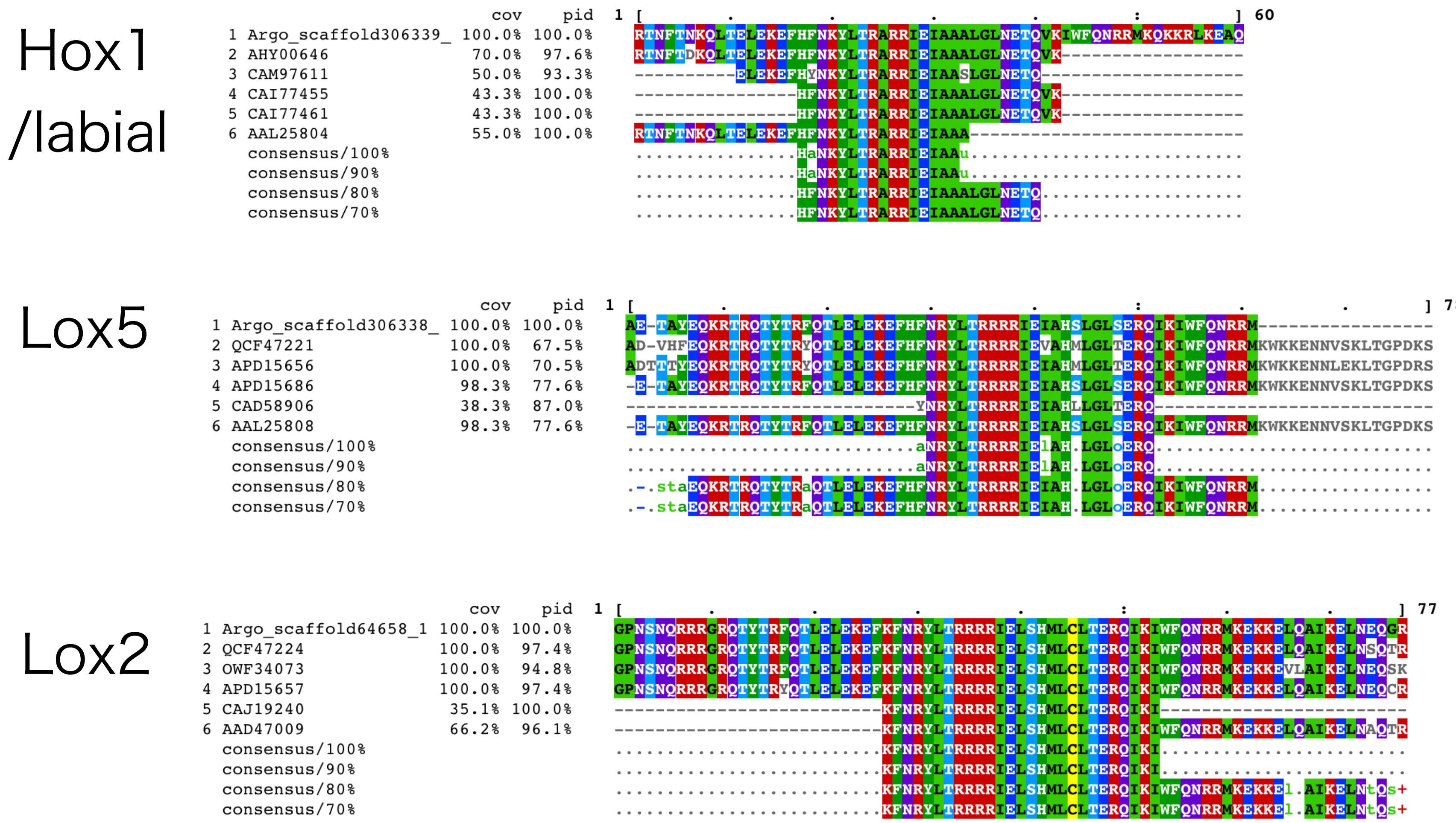

Figure S4 Alignment of Hox genes recovered in the scaffolds but not in the gene models.

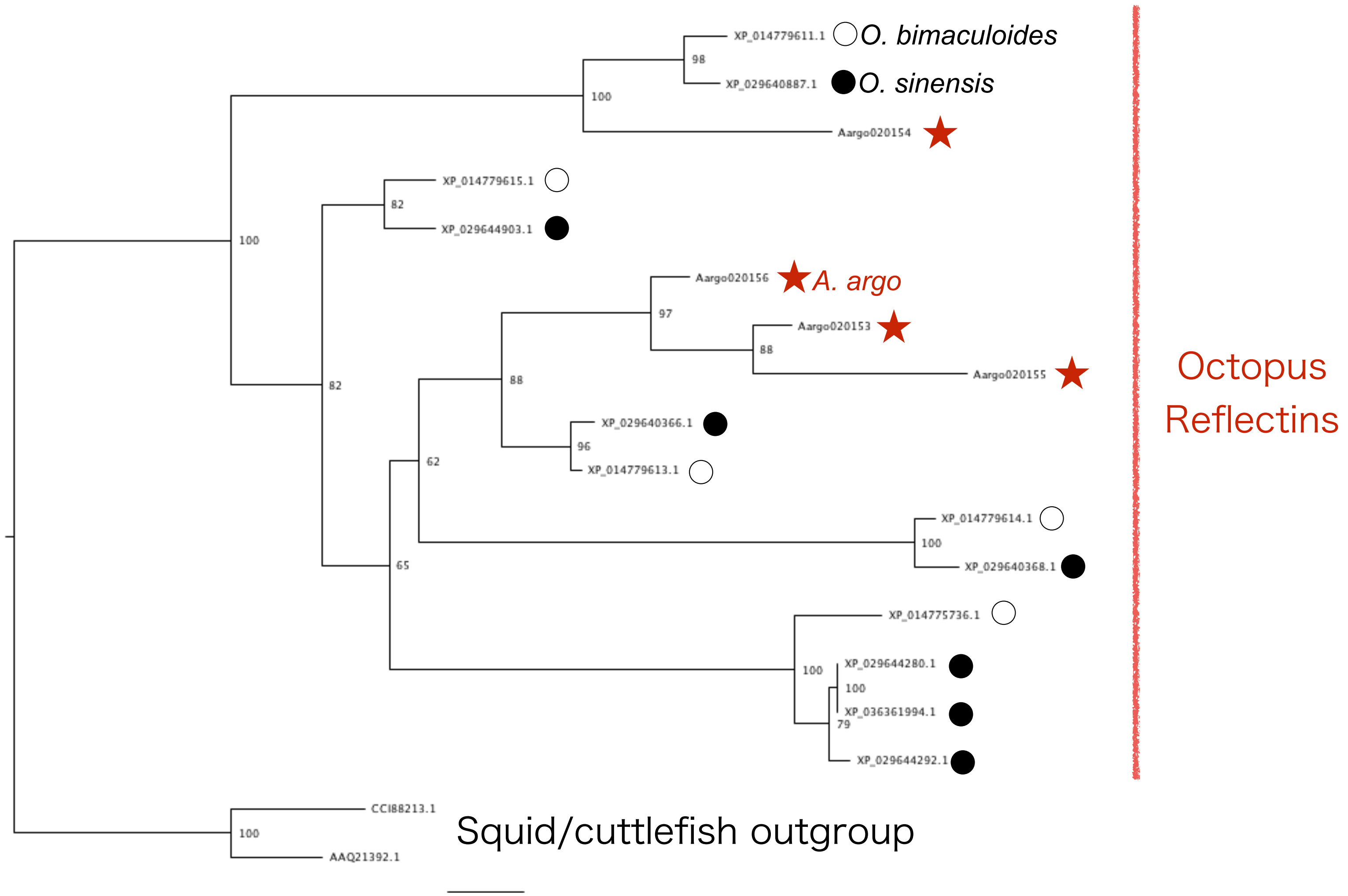

Figure S5 Reflectin phylogenetic tree

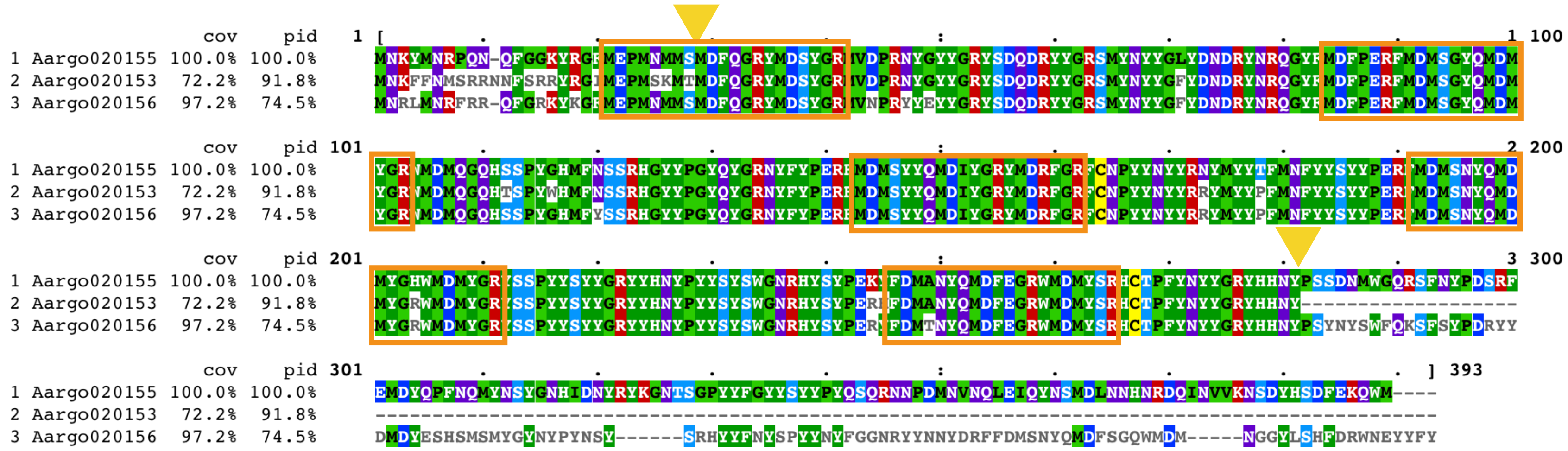

Figure S6 Reflectin alignment with reference to repetitive reflectin motifs

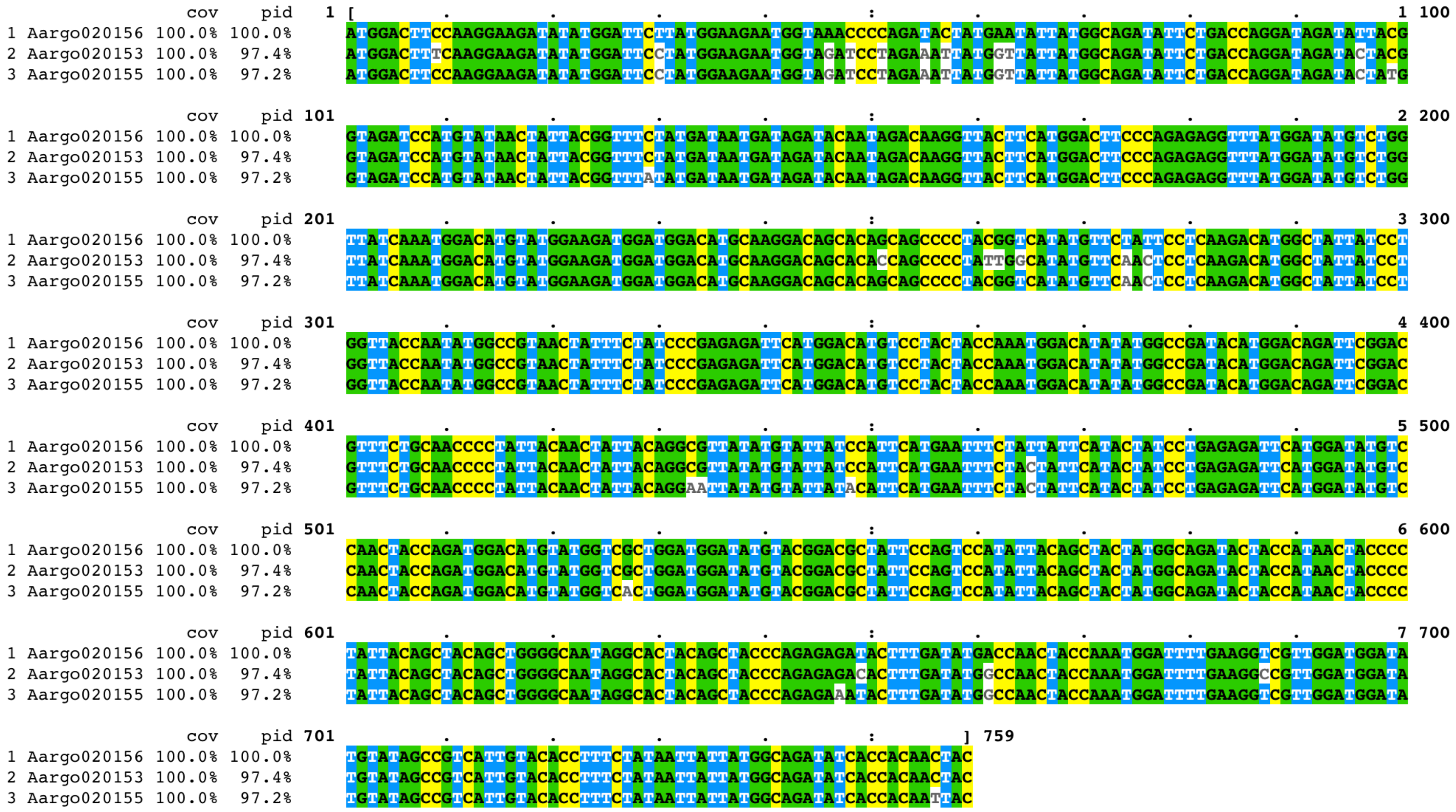

Figure S7 Reflectin alignment to show gene conversion

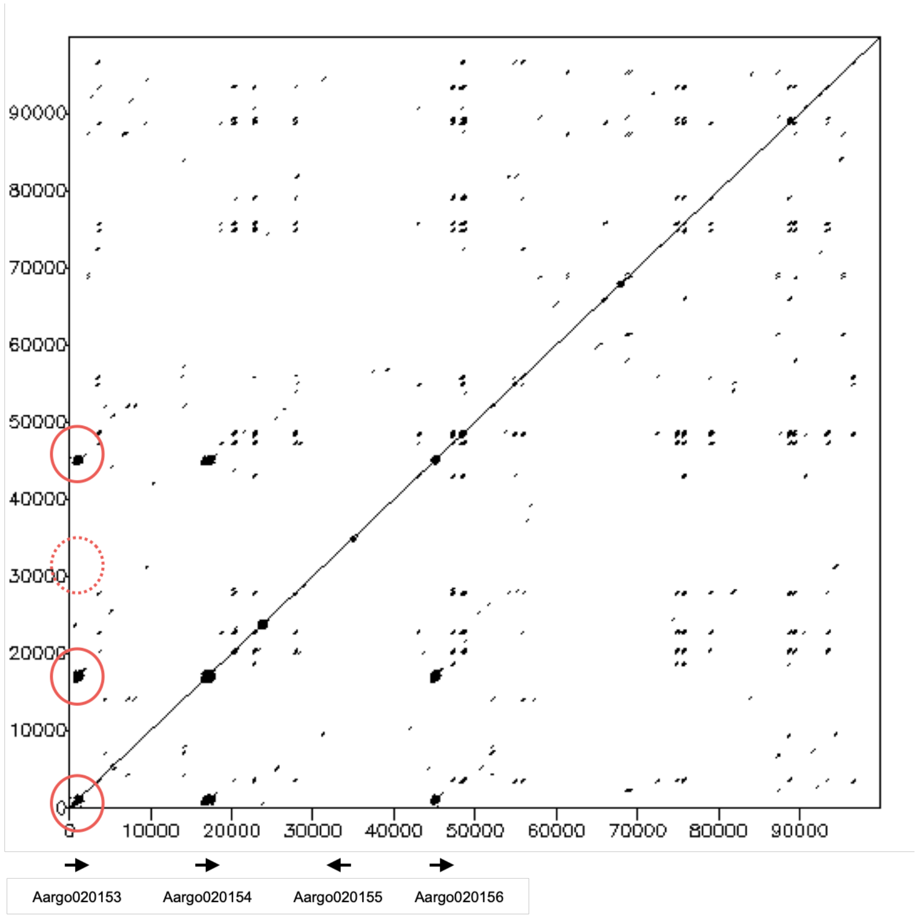

Figure S8 Dot plot analysis of the nucleotide-based alignment of the Reflectin cluster region (own to own alignment of scaffold711789: 14,700,000-14,800,000). Except for the third reflectin copy (Aargo020155, dashed circle), short cluster of homologous regions (red circles), probably reflecting tandemly arranged regions of Reflectin motifs, were observed tandemly.

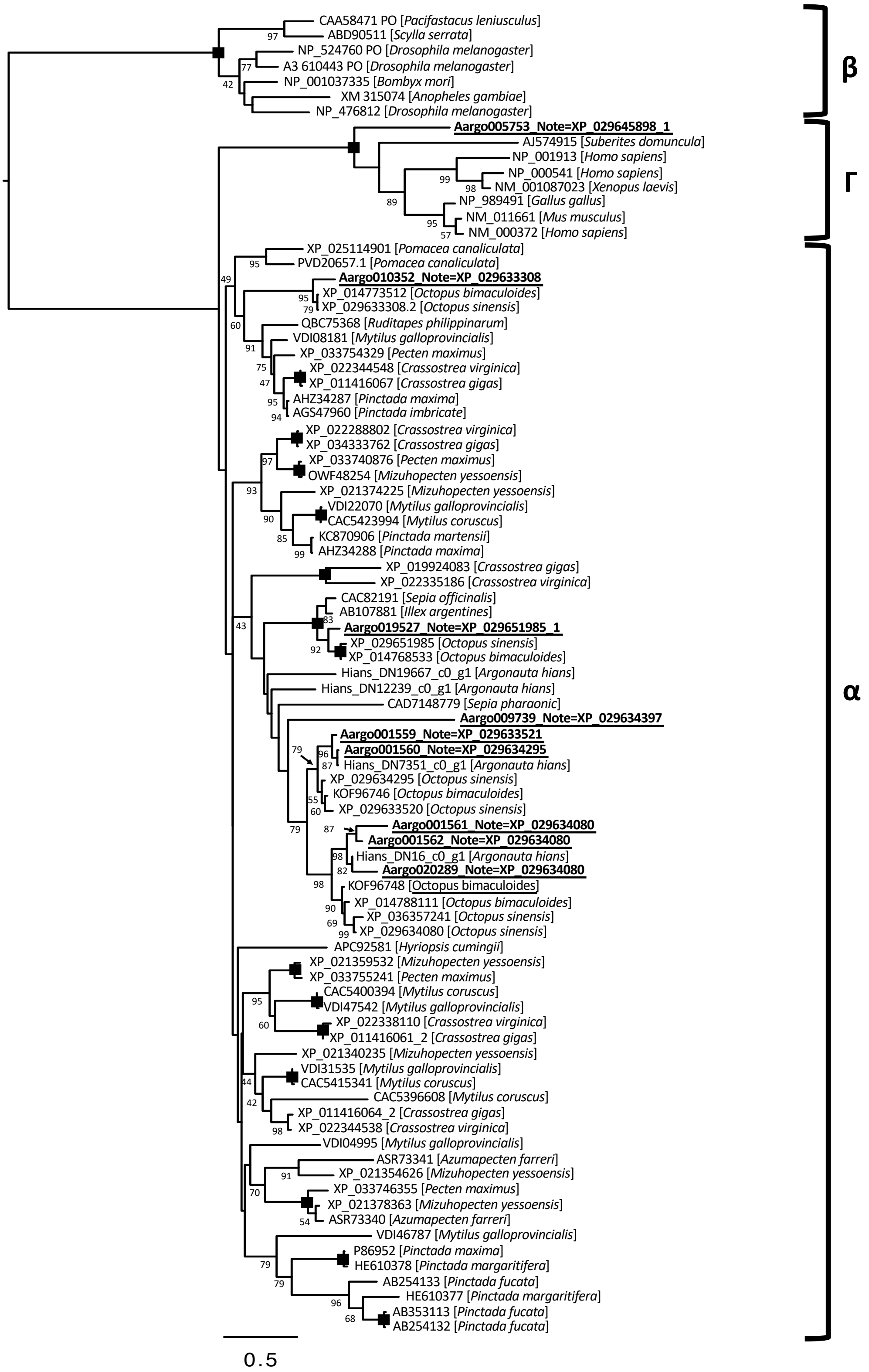

Figure S9 Tyrosinase phylogenetic tree. The maximum likelihood phylogenetic tree

inferred under the LG + Γ + I model with 1000 bootstrap replicates. Numbers on the nodes are Bootstrap Support (BS) values. BS lower than 41% are not shown, while 100% support is shown as a black square. Tyrosinase of *Argonauta argo* are marked with underlined. Three types of tyrosinase are shown secreted (α), cytosolic (β) and membrane-bound (γ) subclasses.

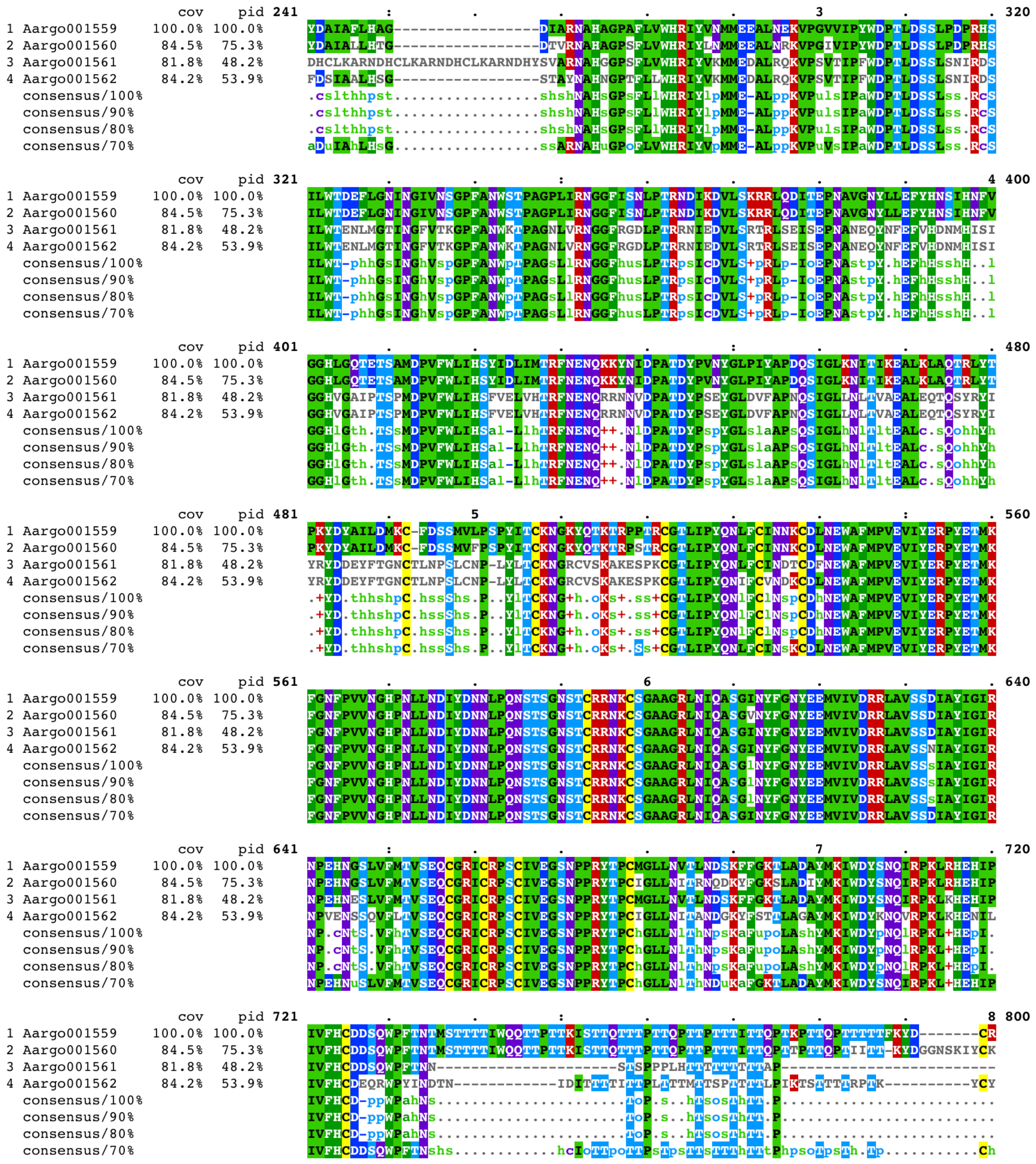

Figure S10 Tyrosinase alignment at amino acid level

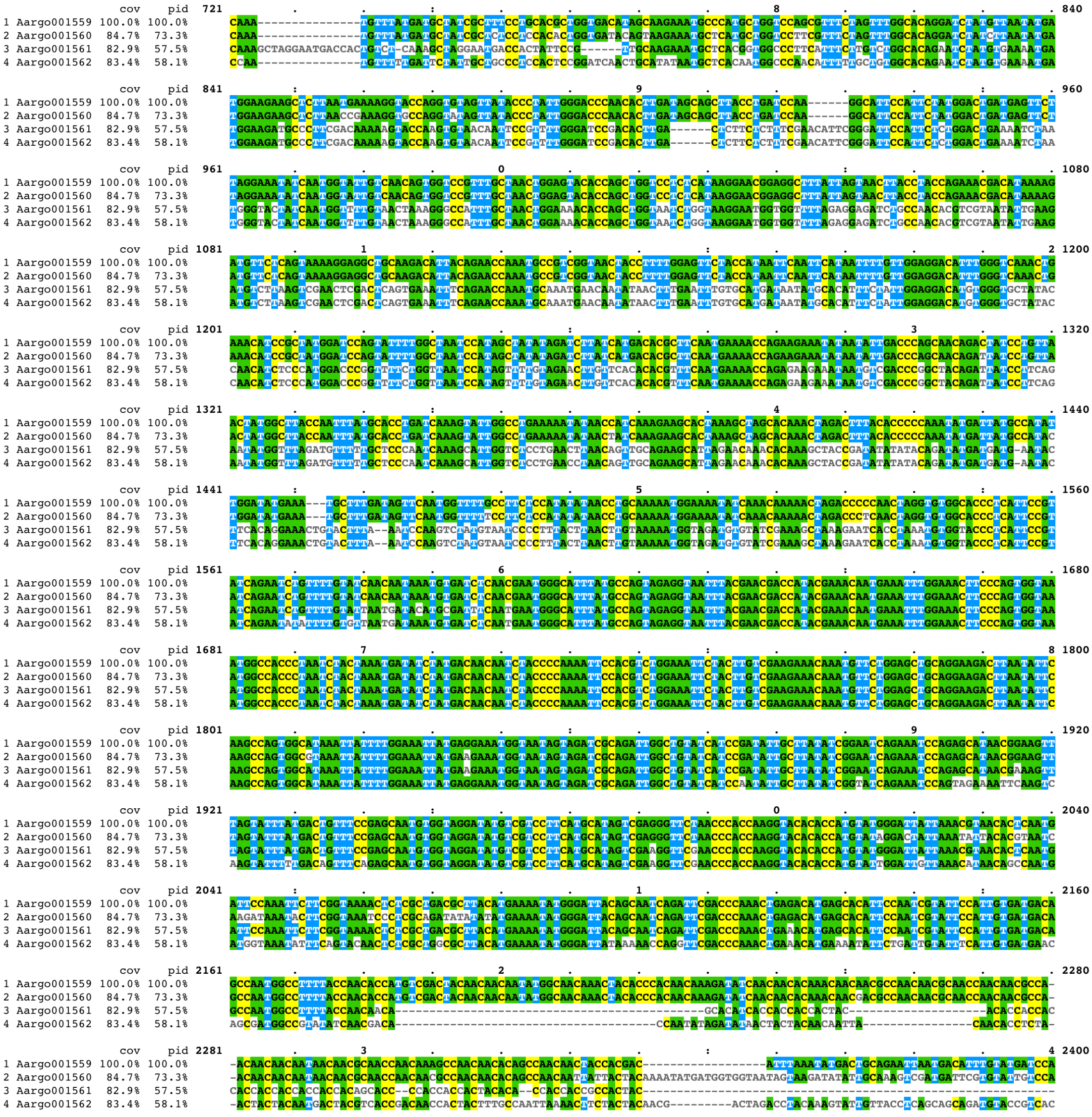

Figure S11 Tyrosinase alignment to show gene conversion

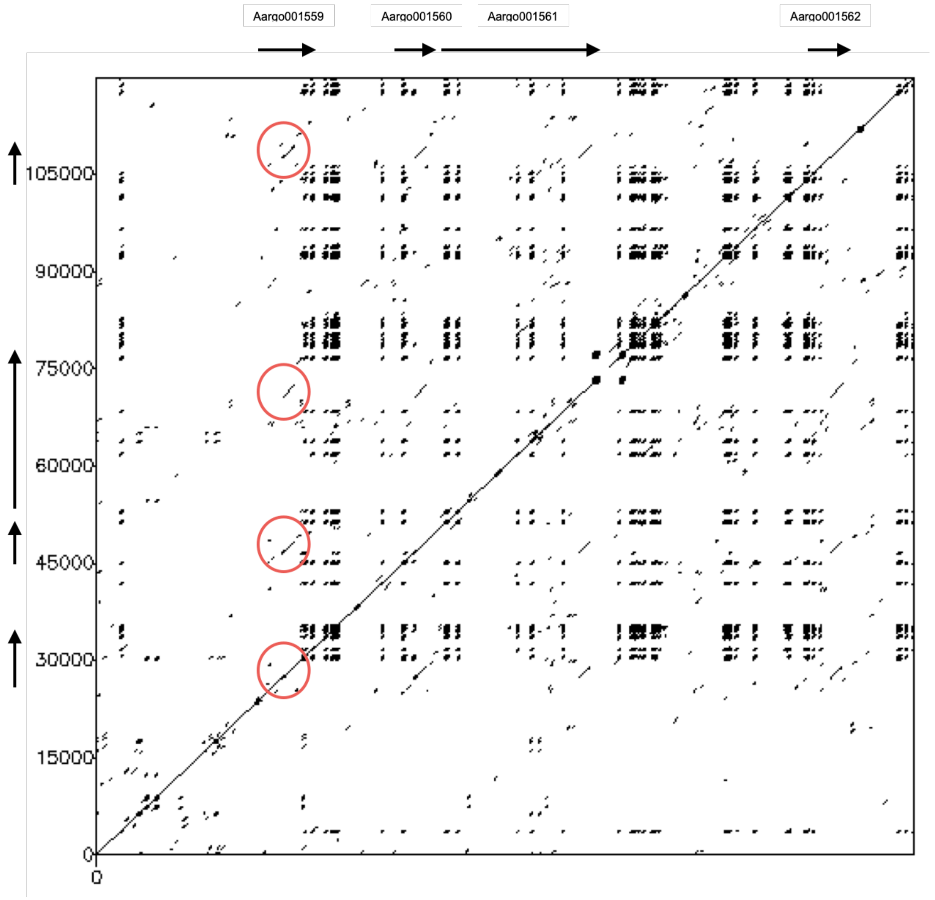

Figure S12 Dot plot analysis of the nucleotide-based alignment of the tyrosinase cluster region (own to own alignment of scaffold36983: 350,000-470,000). Shared sequences extending over 500 bp, possibly beyond a single exon, are shown as shared by four gene regions (red circles). Arrows indicate gene coding regions in this scafflod.

Supplementary notes:

Opsin duplications and change in absorption wavelength might relate to pelagic lifestyle

Changes in the number and sequences of Opsin are thought to be an adaptation to visually guided behavior. We found that *A. argo* possesses five visual pigment genes in its genome: two noncanonical *r-opsins*, one canonical *r-opsin*, two *xenopsin*, and one *rgr/peropsins/retinochromes* (Figure S13-15) (Ramirez et al., 2016). In previous studies on the pygmy squid *Idiosepius paradoxus*, two *r-opsins*, one *xenopsin*, and two *retinochromes* have been identified (Yoshida et al. 2015; Ramirez et al. 2016). We also checked if the expression of the opsins is tissue-specific, in order to see whether there is any functional differentiation among the duplicated opsins. However, at present, we were unable to confirm such specificity, at least in the organs we examined, such as the eye and skin, there is no sufficient expression data. In fact, almost no studies have been conducted on the functions of *xenopsins* and non-canonical *r-opsins* in cephalopods. In the future, a thorough gene expression analysis of these genes in different tissues should be conducted to resolve this issue.

The presence of two xenopsins in the genome of *A. argo* was apparently not an artifact or assembly error, which means that *A. argo* has an extra copy of *xenopsin* than

I. paradoxus. The gene models for *xenopsin* in *A. argo* (Aargo004635 and Aargo004636) exist in tandem in the scaffold, although the amino acid sequence is too short to be considered full-length. If we also assume that the two exons of the neighboring Aargo004633 are shared among the three gene models, we can obtain two putative complete xenopsin proteins. In other words, it is reasonable to think that the two xenopsins were probably splicing variants with alternative promoters and shared two exons, which were then duplicated and subfunctionalized (Force et al., 1999; Hahn, 2009). *Xenopsin* is found to be widespread, but exclusively in protostomes, co-expressed with *r-opsin*, mostly in their ciliary photoreceptor cells (Passamaneck et al., 2011; Vöcking et al., 2017; Rawlinson et al., 2019). Functional studies on *xenopsin* are lacking, and we therefore cannot decisively predict its function in *A. argo*. Phylogenetically, *xenopsin* and *c-opsin* are apparently spread exclusively from each other, with *c-opsin* being found exclusively in the photoreceptor cells of deuterostomes/vertebrates, suggesting that *xenopsin*, similar to its deuterostomian counterpart *c-opsin*. is probably involved mainly in phototactic responses and visual functions in protostomes (Döring et al., 2020), including in *A. argo*, *I. paradoxus*, and probably other cephalopods.

The two copies of non-canonical *r-opsins* in the genome of *A. argo* are most likely

due to the duplication of heterogeneous regions in the assembly, since the sequences matched perfectly. This suggests that there is only a single non-canonical *r-opsin* in the genome of *A. argo*, which is similar to *I. paradoxus*, as mentioned previously (Yoshida et al. 2015). At present, the function of this opsin homolog is still unknown, although previous studies suggest that it is probably unrelated to vision, although apparently still related to photoreception (Ramirez and Oakley, 2015; Ramirez et al. 2016; Bonade et al., 2020). We found two amino acid substitutions (T118S and Y178F) in the amino acid sequence of the non-canonical r-opsin of *A. argo* when compared to bovine rhodopsin. T118S was found in both benthic *O. bimaculoides* and *A. argo*, while Y178F was found only in the latter. The prediction of the light absorption wavelength of the non-canonical r-opsin of *A. argo* indicates that photoreceptions in *A. argo* are probably adapted more to red light than that of the benthic octopus. The extra amino acid substitutions are thus consistent with the ecology of *A. argo*, which lived closer to the sea surface than other cephalopods, indicating that the red-shift may be an adaptation to a shallow water light environment.

References

Bonadè M, Ogura A, Corre E, Bassaglia Y, Bonnaud-Ponticelli L. 2020. Diversity of Light

Sensing Molecules and Their Expression During the Embryogenesis of the Cuttlefish (*Sepia officinalis*). Front. Physiol. 11:521989. doi: 10.3389/fphys.2020.521989.

Döring CC, Kumar S, Tumu SC, Kourtesis I, Hausen H. 2020. The visual pigment xenopsin is widespread in protostome eyes and impacts the view on eye evolution. Elife. 9. doi: 10.7554/eLife.55193.

Force A et al. 1999. Preservation of duplicate genes by complementary, degenerative mutations. Genetics. 151:1531–1545. doi: 10.1093/genetics/151.4.1531.

Hahn MW. 2009. Distinguishing among evolutionary models for the maintenance of gene duplicates. J. Hered. 100:605–617. doi: 10.1093/jhered/esp047.

Passamaneck YJ, Furchheim N, Hejnol A, Martindale MQ, Lüter C. 2011. Ciliary photoreceptors in the cerebral eyes of a protostome larva. Evodevo. 2:6. doi: 10.1186/2041-9139-2-6.

Ramirez MD et al. 2016. The Last Common Ancestor of Most Bilaterian Animals Possessed at Least Nine Opsins. Genome Biol. Evol. 8:3640–3652. doi: 10.1093/gbe/evw248.

Ramirez MD, Oakley TH. 2015. Eye-independent, light-activated chromatophore expansion (LACE) and expression of phototransduction genes in the skin of *Octopus bimaculoides*. J. Exp. Biol. 218:1513–1520. doi: 10.1242/jeb.110908.

Rawlinson KA et al. 2019. Extraocular, rod-like photoreceptors in a flatworm express xenopsin photopigment. Elife. 8. doi: 10.7554/eLife.45465.

Vöcking O, Kourtesis I, Tumu SC, Hausen H. 2017. Co-expression of xenopsin and rhabdomeric opsin in photoreceptors bearing microvilli and cilia. Elife. 6. doi: 10.7554/eLife.23435.

Yoshida MA et al. 2015. Molecular Evidence for Convergence and Parallelism in Evolution of Complex Brains of Cephalopod Molluscs: Insights from Visual Systems. Integr. Comp. Biol. 55:1070–1083. doi: 10.1093/icb/icv049.

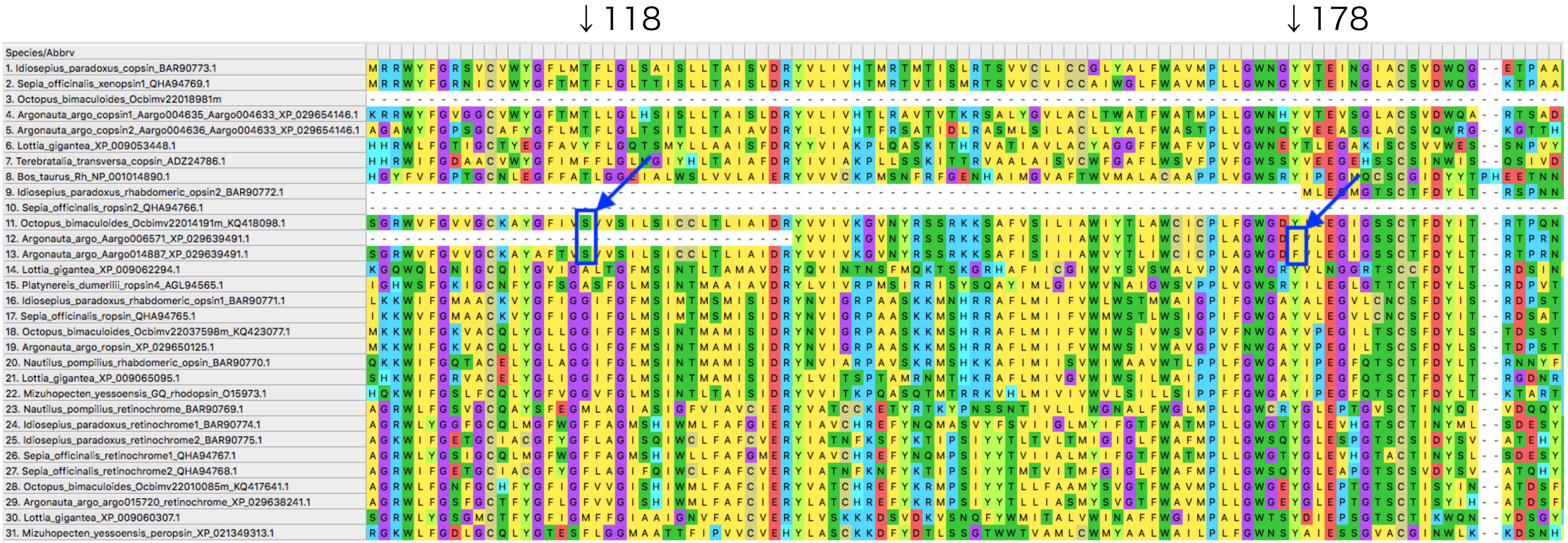

Figure S13 Opsin alignment and unique amino acid changes of argonaut based on bovine rhodopsin

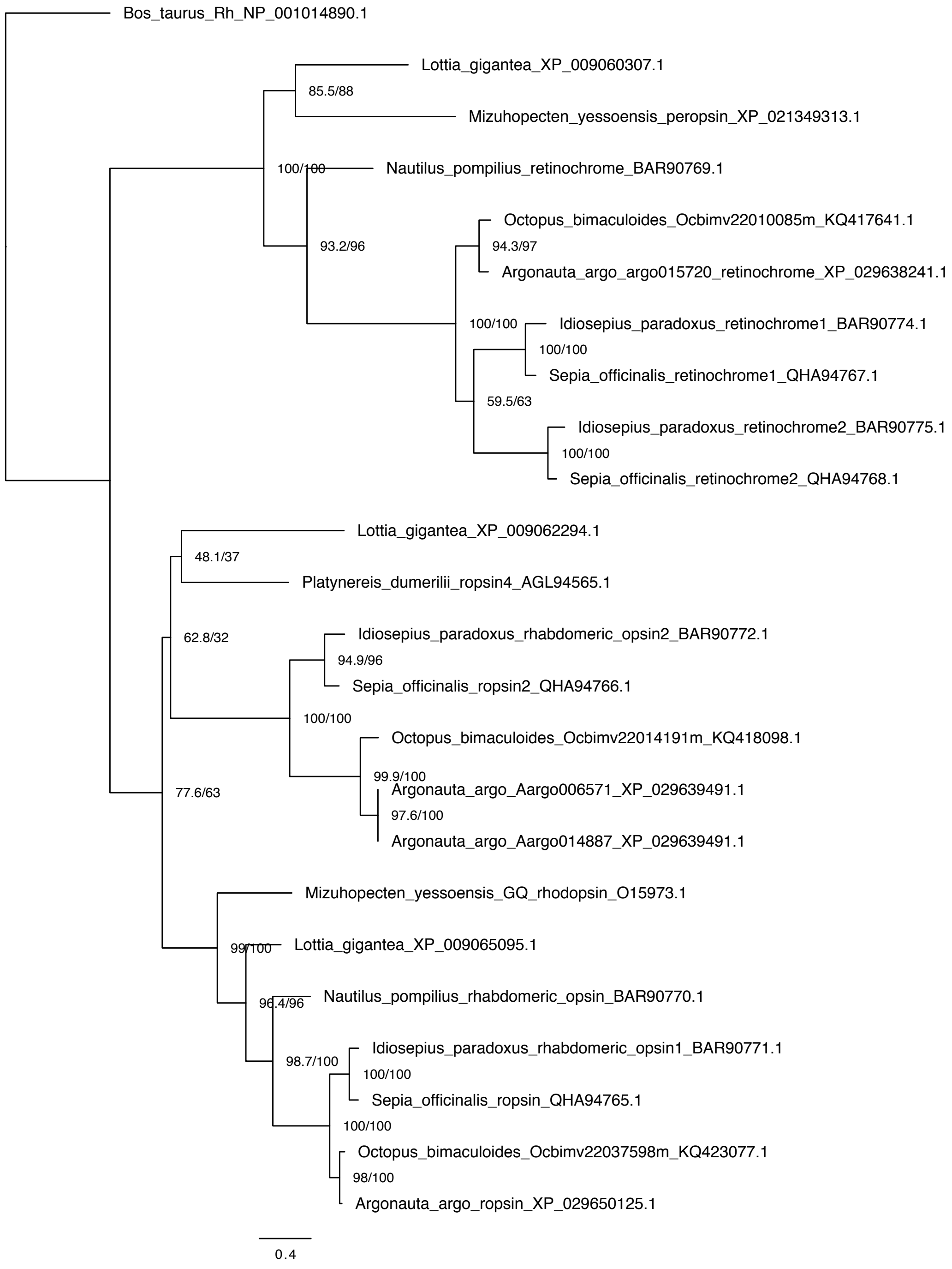

Figure S14 Phylogenetic tree of RGR and rhabdomeric opsins

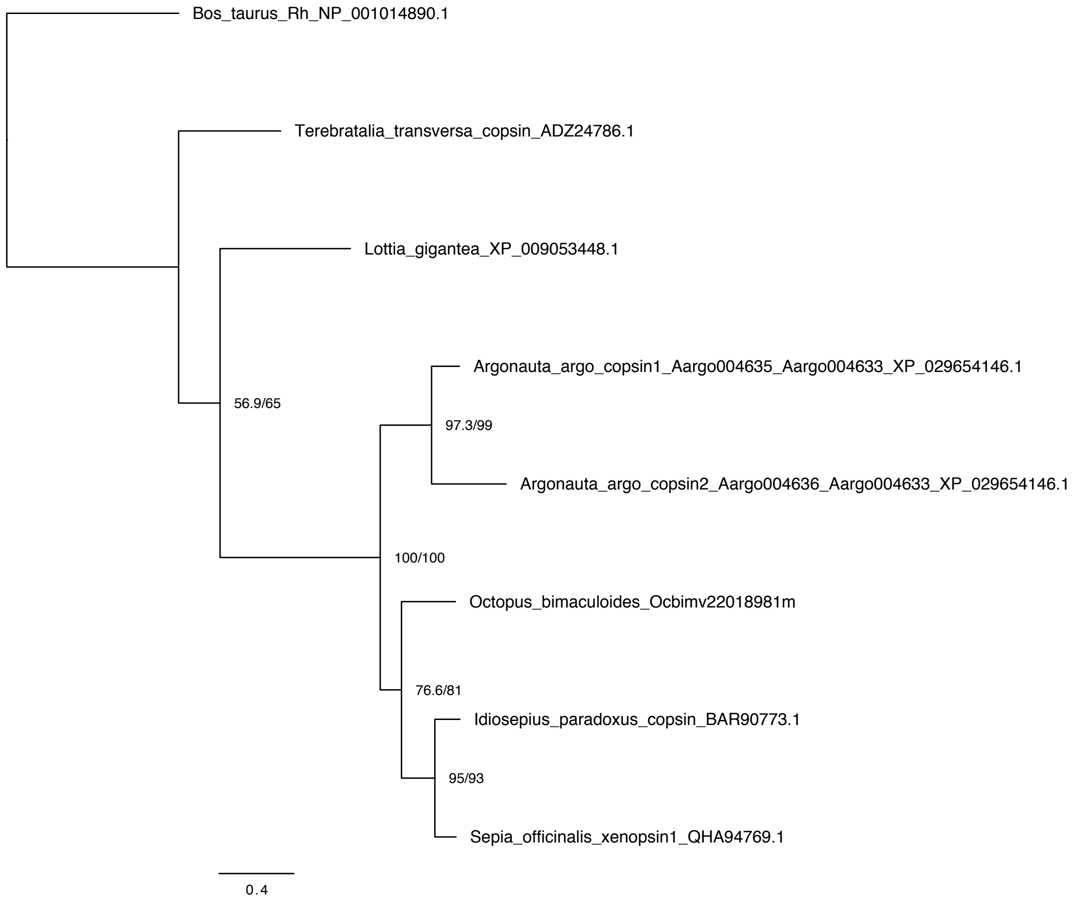

Figure S15 Xenopsin phylogenetic tree
